# Deciphering the mechanisms, hormonal signaling, and potential applications of endophytic microbes to mediate stress tolerance in medicinal plants

**DOI:** 10.3389/fpls.2023.1250020

**Published:** 2023-11-15

**Authors:** Praveen Pandey, Arpita Tripathi, Shweta Dwivedi, Kanhaiya Lal, Tripta Jhang

**Affiliations:** ^1^ Microbial Technology Department, CSIR-Central Institute of Medicinal and Aromatic Plants, Lucknow, India; ^2^ Division of Plant Breeding and Genetic Resource Conservation, CSIR-Central Institute of Medicinal and Aromatic Plants, Lucknow, India; ^3^ Faculty of Education, Teerthanker Mahaveer University, Moradabad, India; ^4^ Academy of Scientific and Innovative Research (AcSIR), Ghaziabad, India

**Keywords:** plant-microbe interaction, medicinal plants, biotic-abiotic stress, signaling pathways, ethylene, salicylic acid, jasmonic acid

## Abstract

The global healthcare market in the post-pandemic era emphasizes a constant pursuit of therapeutic, adaptogenic, and immune booster drugs. Medicinal plants are the only natural resource to meet this by supplying an array of bioactive secondary metabolites in an economic, greener and sustainable manner. Driven by the thrust in demand for natural immunity imparting nutraceutical and life-saving plant-derived drugs, the acreage for commercial cultivation of medicinal plants has dramatically increased in recent years. Limited resources of land and water, low productivity, poor soil fertility coupled with climate change, and biotic (bacteria, fungi, insects, viruses, nematodes) and abiotic (temperature, drought, salinity, waterlogging, and metal toxicity) stress necessitate medicinal plant productivity enhancement through sustainable strategies. Plants evolved intricate physiological (membrane integrity, organelle structural changes, osmotic adjustments, cell and tissue survival, reclamation, increased root-shoot ratio, antibiosis, hypersensitivity, etc.), biochemical (phytohormones synthesis, proline, protein levels, antioxidant enzymes accumulation, ion exclusion, generation of heat-shock proteins, synthesis of allelochemicals. etc.), and cellular (sensing of stress signals, signaling pathways, modulating expression of stress-responsive genes and proteins, etc.) mechanisms to combat stresses. Endophytes, colonizing in different plant tissues, synthesize novel bioactive compounds that medicinal plants can harness to mitigate environmental cues, thus making the agroecosystems self-sufficient toward green and sustainable approaches. Medicinal plants with a host set of metabolites and endophytes with another set of secondary metabolites interact in a highly complex manner involving adaptive mechanisms, including appropriate cellular responses triggered by stimuli received from the sensors situated on the cytoplasm and transmitting signals to the transcriptional machinery in the nucleus to withstand a stressful environment effectively. Signaling pathways serve as a crucial nexus for sensing stress and establishing plants’ proper molecular and cellular responses. However, the underlying mechanisms and critical signaling pathways triggered by endophytic microbes are meager. This review comprehends the diversity of endophytes in medicinal plants and endophyte-mediated plant-microbe interactions for biotic and abiotic stress tolerance in medicinal plants by understanding complex adaptive physiological mechanisms and signaling cascades involving defined molecular and cellular responses. Leveraging this knowledge, researchers can design specific microbial formulations that optimize plant health, increase nutrient uptake, boost crop yields, and support a resilient, sustainable agricultural system.

## 1 Introduction

Medicinal plants are crucial in the pharmaceutical and drug industries for providing many pharmaceutically vital bioactive molecules for herbal medicine. Rising consumer demand for herbal drugs and natural products has significantly increased the cultivation acreage of medicinal plants, competing with fixed land resources for cereals and other horticultural crops. The intent of increasing productivity per unit area from the limited land resources has led to excessive usage of agrochemicals (fertilizers, insecticides, pesticides, weedicides, etc.) consumption over the past few decades. Their redundant usage has critically affected soil microbiome and environmental health. Therefore, developing green, efficient, affordable, and eco-friendly agrotechnologies is essential for improving medicinal plants’ health and productivity. Sustainable agricultural production is a significant challenge in the global climate change paradigm. In this context, harnessing endophytic microbes as biostimulants can be an effective, sustainable approach. Endophytes are microorganisms (bacteria or fungi) that spend at least a portion of their life cycle forming an association with an asymptomatic plant (Vanessa and Christopher, 2004). Medicinal plants are strongly influenced by microbial endophyte association. In general, endophytic microbes can modify their structure and diversity depending on genotypes, organs, health conditions, and growth stages of host medicinal plants in order to obtain a constant supply of nutrients. Medicinal plants have a range of physiological characteristics, metabolites, and growth patterns that influence their ability to attract different endophytic microbes. Environmental factors considerably impact the quality and yield of medicinal plants. They not only affect the distribution of a medicinal plant but also determine the species of microbial endophytes that can colonize the host during its life cycle.

Plants grown in biologically diverse soil abundant with beneficial microbes have better survival under harsh conditions. The plant’s roots anchor it to the soil, enabling it to absorb minerals and essential nutrients and synthesize chemical substances mediating various plant-microbe interactions. These interactions comprise mutualistic relationships with beneficial microbes; however, parasitism occurs with harmful microbes (Badri et al., 2009). The plant deploys surface-localized receptor proteins to recognize self-modified or microbe-derived molecules to recognize microbial invaders are potentially harmful or beneficial microbes. The recognition of β-glucan chains and plant immunity depends on the degree of polymerization and β-1,3-glucan receptor systems perception by a specific plant species (Wanke et al., 2020). The positive interactions have practical implications useful in pharmaceutical, biotechnological, and agricultural applications, but the negative interactions lead to severe plant diseases that endanger global agricultural productivity. Utilizing plant-microbe interactions eliminates the need for synthetic inorganic pesticides and fertilizers, which lowers input costs and, thus, minimizes the impact of synthetic agrochemicals on vital existing ecological communities (Whipps and Gerhardson, 2007). Furthermore, plant-microbe symbiosis produces crucial compounds of industrial and pharmaceutical interest, which eliminates the need for costly catalysts and synthetic derivatives (Wu et al., 2007).

Integrating plant-associated microbes into farming to support agricultural production mitigates a series of biotic and abiotic perturbations (Tanaka et al., 2005; Vega et al., 2008; Wani et al., 2016; Lata et al., 2018; Mukherjee et al., 2021 **;** Siddique et al., 2022). Biotic and abiotic factors influence many morpho-physiological disturbances in plants, including stunted growth and development, senescence, altered gene expression, cellular metabolism, etc., reducing overall crop yield and quality (Purohit et al., 2019). Abiotic stresses are caused by non-living factors such as drought, salinity, waterlogging, temperature extremes (heat, cold, and freezing), metal toxicity, etc., while biotic stresses (caused by living organisms, especially bacteria, fungi, viruses, insects, nematodes, and weeds, etc.), directly starve the hosts of their nutrients limiting the growth or plant death resulting in the pre- and post-harvest crop losses. Plants can mitigate biotic stressors even if they lack an adaptive immune system by adjusting to specific, sophisticated strategies such as antibiosis, hypersensitivity, allelochemical synthesis, membrane integrity, organelle modifications, etc. Plants’ genetic makeup controls the defensive schemes that respond to these stresses. Numerous genes in the plant genome are either tolerant or resistant to various biotic stressors. Being sessile, plants have no choice to escape these environmental cues; however, they alter their genetic architecture for stress adaptation. Specifically, by inducing immunological responses, generating antioxidants, and inhibiting pathogen growth, endophytic microorganisms help plants cope with biotic and abiotic stress. Notably, the interaction between plants and microbes results in the production of a wide range of bioactive substances, including artemisinin, taxol, phenolic acid, huperzine, azadirachtin, vindoline, guanosine, inosine, serpentine ajmalicine, curcumin, and camptothecin, which are profoundly utilized in agriculture and medicine.

Endophytes modulate levels and activity of phytohormones, viz., gibberellins, cytokinins, ethylene (ET), abscisic acid (ABA), jasmonic acid (JA), and salicylic acid (SA), which play a crucial role in plant growth, fitness, and stress amelioration (Barnawal et al., 2016; Egamberdieva et al., 2017; Xu et al., 2018; Sabagh et al., 2021; Chaudhary et al., 2022; Tripathi A. et al., 2022). In stressful conditions, plant defense systems trigger appropriate cellular responses by responding to stimuli from sensors situated on the cytoplasm or cell surface and transmitting signals to the transcriptional machinery in the nucleus with the help of various signaling pathways. Signaling pathways are crucial for sensing stress and establishing the proper molecular and cellular responses (Mir et al., 2022). Phytohormones are an integral part of the plant defense system, commonly known as the plant’s systemic acquired resistance (SAR) and induced systemic resistance (ISR). These plant hormones operate as plant protective agents against different phytopathogens. In addition to regulating plant physiological and morphological responses, phytohormones also shape the plant microbiome. Different phytohormones induce distinct effects on plant microbiomes. Plants constantly face a wide range of biotic and abiotic stresses that lead to specific transcriptional variations at the individual gene level, with high variability and stress specificity. Therefore, more practical and fundamental studies are required to address the processes and functioning of hormonal signaling and crosstalk. Hence, this review focuses on a detailed overview of the diversity of endophytes in medicinal plants and defense mechanisms at the cellular level associated with endophyte-mediated plant-microbe interactions for biotic-abiotic stress alleviation, including different signaling pathways.

## 2 Diversity of endophytic microbes in medicinal plants

**Figure 1:**
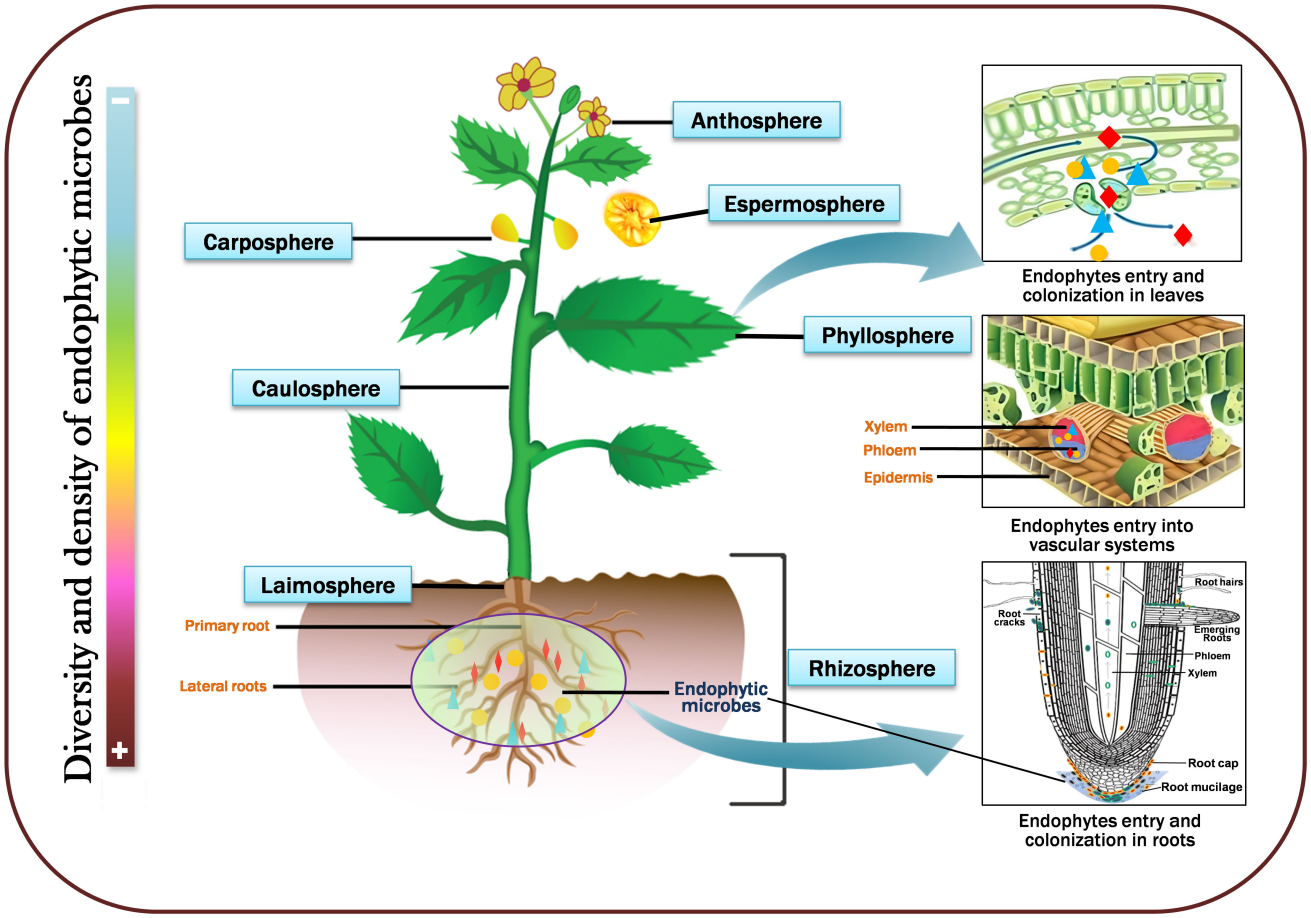
A simplified diagramm showing microbial diversity in various plant parts viz., leaves (phyllosphere), above ground stem (caulosphere), below ground stem (laimosphere), flowers (anthosphere), fruits (carposphere), and seeds (spermasphere). Sidebar color intensities represent microbial density and diversity; dark red represents high, and light blue indicates low diversity and density.

Endophytic microbes live in various plant habitats that communally shape the plant endomicrobiome and are most frequently found in plant roots, stems, leaves, fruits, and seeds. Generally, they establish communities in intercellular spaces; nevertheless, certain species can penetrate cells (Toubal et al., 2018). The primary habitat and colonization of endophytic microbes are roots, and their preferred entry points are root hairs, cracks, or wounds caused by phytopathogen infection; this permits the leakage of metabolites that attract more endophytes. Nevertheless, the other vital regions for root colonization are the cortex and epidermis intercellular gaps (Compant et al., 2005). For instance, the root colonization of *Piriformospora indica*, commences in the cortical area with a biotrophic development stage and proceeds to a cell death-dependent step. Rhizospheric microbes associated with Fenugreek (*Trigonella foenumgraecum*) stimulate host plant growth via soil nutrient uptake and recycling (Kumari et al., 2020). Different endophytes may serve as the primary root mutualistic symbionts in stressful situations where mycorrhizae are often scarce (Mandyam et al., 2010; Rat et al., 2021). Sometimes, endophytes enter within the xylem vessels that migrate from the root zones; several harbor-diversified communities penetrate the aerial regions utilizing the soil surface. The majority of endophytic microorganisms embrace an array of entryways, especially the leaves (phyllosphere), above ground stem (caulosphere), below ground stem (laimosphere), flowers (anthosphere), fruits (carposphere), and seeds (spermasphere) (Lindow and Brandl, 2003; Ritpitakphong et al., 2016; Abdullaeva et al., 2020 **;** Sun et al., 2023). Upon arriving leaves and stems from openings like stomata, they grow and create a thin biofilm (Frank et al., 2017). In addition, several microbes might penetrate the inner regions and establish where other microorganisms may invade the xylem. They continue to colonize and grow in various organs, such as the caulosphere, phylloplane, anthrosphere, and carposphere (Meyer and Leveau, 2012). These microbes are inherently advantageous in that they serve as a marker for the beginning of the community structure in the seedling and the end of the community assemblage in the seed (Shahzad et al., 2018). They are pretty intriguing since they transmit their personalities to subsequent generations vertically and can generate endospores, uphold plant growth, control cell motility, and regulate endogenous phytohormones, which improve the structure of the soil, disrupt seed dormancy, and degrade xenobiotics. However, seed endophytes developed multiple paths; few penetrate through the xylem, stigma, and the extrinsic route, wherein an external factor contaminates seeds. The floral components of plants have not been comprehensively investigated to study endophytic diversity; nevertheless, Qian et al. (2014) isolated an endophytic fungus, *Lasiodiplodia* sp., from floral parts of *Viscum coloratum*, which is involved in the synthesis of vital metabolites. Therefore, the diversity of endophytic communities is primarily determined by a series of transforming factors, including the host genetic makeup and immune system, the environment, microbe-microbe interactions, types of soil, and nutrition. **Figure 1** depicts the schematic representation of the diversity of endophytic microbes in various plant parts.

## 3 The complexity of the plants-microbes relationship

Plant-microbe interactions bear a complex relationship depending on the biological and physicochemical ecology of soil, seed surface, phyllosphere, and rhizosphere. While “obligate” microbes interact with living cells in order to develop and complete their life cycle, “epiphytes” grow upon another plant merely for physical support, and “opportunistic” microbes occasionally penetrate the endosphere of plants (Hardoim et al., 2008). The plant and the endophyte coexist in this interaction and greatly benefit one another (Ting et al., 2009). These endophytes are frequently rhizospheric; basal root zones with tiny crevices and the apical root zone may be the ideal sites for their linkage and subsequent entrance into the host (Gagne et al., 1987). They multiply throughout the host plant (Hallmann et al., 1997) and dwell in the cells, vascular system, or intercellular regions (Bell et al., 1995). While roots have the most excellent chance of colonization through the epidermis created by the lateral root system, endophytic microbes could penetrate through the stomata and transmit vertically to offspring via maternal seeds (Agarwal and Shende, 1987). It is indisputable from the “balanced antagonism” during asymptomatic colonization among the host and endophytic microorganisms that endophytes can survive inside the host without invoking any innate immunity and enhance their ability to sustain themselves by producing substances that are similar to those of plants (Schulz and Boyle, 2005). According to extensive research on the symbiotic association between endophytic microbes and their host plant, the plant safeguards and sustains the endophytes, which ‘in return’ deliver natural compounds with therapeutic potential (antiviral, antifungal, antibacterial, insecticidal, etc.) to uplift the former’s productivity and sustainability in their natural habitat. Additionally, they defend host plants from phytopathogens by triggering the synthesis of plant secondary metabolites under adverse conditions (Azevedo et al., 2000; Strobel, 2003). Hence, they are now considered an essential component of biodiversity; the distribution of endophytic microflora varies depending on the host. They have been found inside nearly all vascular plants, notably those with medicinal properties that have been assumed to be linked to drug synthesis; several studies have shown that these endophytes represent a significant source of medicinal compounds (Zhang et al., 2006).

Endophytic microbes have a wide and diverse niche in plants, which leads to a complex relationship that implies mutualism, antagonism, and rarely parasitism (Nair and Padmavathy, 2014). They reside within the plant tissue, wherein numerous bacteria and fungi species constitute the “plant endomicrobiome,” capable of triggering a number of cellular and physiological changes in the plant. Some relationships between plants and microbes are commensalism, whereby the plant incurs no harm, but the microbe benefits. The microbes and the plant interact through chemical signaling molecules released by the plants and discharge of corresponding microbial substances (phenols, steroids, taxol, xanthones, terpenoids, benzopyranones, isocoumarins, chinones, tetralones, cytochalasins, and enniatines, etc.), resulting in a two-way “crosstalk” that employs signal transduction. Once a link between plant and microbe is established, both organisms continue to monitor each others’ physiology and adjust their behavior accordingly. Endophytic bacteria have a considerable advantage over plants’ rhizospheric bacteria and provide more benefits than microorganisms outside of the plants and in the rhizosphere because they are in direct contact with the plant tissues (Araujo et al., 2002; Hardoim et al., 2015). Fungal endophytes spread into progeny via hyphal fragments or spores in above-ground tissues by pathogens (biotic dispersal agents) or air or water (abiotic dispersal agents) through parent plants, whereby the progeny become infected (Hodgson et al., 2014; Gagic et al., 2018), growing in the rhizosphere’s nutrient-rich environment, harboring airborne pathogenic organisms (Sasse et al., 2018), enabling transmission of fungal endophytes across different host species (Wiewiora et al., 2015).

## 4 Interaction of secondary metabolites of the host and metabolites from endophytic microbes

The interaction between secondary metabolites of the host and metabolites from endophytic microbes is a complex and dynamic process that can result in diversified effects from beneficial to detrimental. One of the most fascinating aspects of endophytic microbes is their potential to synthesize bioactive compounds that might interact with secondary metabolites of their host. Plant secondary metabolites perform diverse functions in plants, including growth and development, inherent immunity (Piasecka et al., 2015), defense responses (Isah, 2019), stress adaptation (Yang et al., 2018), phytopathogen control, operating as signals for plant-microbe symbiosis, and transforming microbial communities linked to hosts (Guerrieri et al., 2019). Similarly, plant microbiomes are involved in many of the abovementioned processes, directly or indirectly modulating plant metabolism (Trivedi et al., 2020; Adeleke and Babalola, 2021; Ayilara et al., 2022). Plants can shape their microbiome by secreting an array of metabolites; consequently, the microbiome could affect the host plants’ metabolome. Perhaps in medicinal plants, the stimulation of secondary metabolites through endophytes is a common phenomenon that can transform the rhizobiome (Sasse et al., 2018; Cotton et al., 2019). Recent research suggested that interactions between plants and their microbiomes could increase the biomass of *Salvia miltiorrhiza*, having a unique microbiome (*Sphingomonas, Pantoea, Dothideomycetes*, and *Pseudomonas*), as well as affect the synthesis of a novel bioactive compound “tanshinone” (Chen et al., 2018; Huang A. C. et al., 2019). Similarly, *Marmoricola* sp. and *Acinetobacter* sp. enhanced morphine content in *Papaver somniferum* via modulating expression of morphine biosynthesis genes (Ray et al., 2019), and *Phialemoniopsis cornearis, Fusarium redolens*, and *Macrophomina pseudophaseolina* influenced forskolin biosynthesis in a medicinal plant *Coleus forskohlii* (Mastan et al., 2019). Using a chemical recognition framework, plants can also recognize specific molecules released by microbiomes that trigger plants to build signaling networks, modify associated gene functions, and accumulate specific secondary metabolites (Tidke et al., 2019). Nevertheless, it is likely that a portion of these so-called “secondary metabolites” are actually the metabolic by-products of their endophytic microbes. Endophytic microbes can synthesize numerous secondary metabolites, such as paclitaxel (taxol), podophyllotoxin, camptothecin, and deoxypodophyllotoxin, which are also generated by plants (Etalo et al., 2018; Furtado et al., 2019; Pang et al., 2021). Consequently, it is crucial to distinguish which metabolites originated from the plant microbiome and which ones from the host.

The effects of microbial secondary metabolites on plants have been well-documented. Even though some pathogenic microbes secrete toxins that harm plants, such as fumonisins and AAL-toxins made by the *Fusarium* sp. and *Alternaria alternata f.* sp. (Chen et al., 2020), many microbes synthesize valuable secondary metabolites that promote plant growth; for example, *Bacillus tequilensis* SSB07 produces several phytohormones viz., gibberellins, IAA, and ABA which boosted growth and thermotolerance in soybean (Kang et al., 2019). Plant microbiomes can also produce numerous volatile organic compounds (aldehydes, alcohols, ammonia, ketones, terpenes, esters, etc.) that can influence plant development, communication, pathogen defense, and prevent herbivorous insects and parasitic nematodes (Kai et al., 2009; Ortíz-Castro et al., 2009; Zhang et al., 2020). Maggini et al. (2017) reported that the influence of the interaction between the medicinal plant *Echinacea purpurea* (L.) Moench and its endophytic microbes revealed that microbes could affect the synthesis of volatile organic compounds, phenylpropanoid, and alkamides in the host. Besides, plant-derived non-volatile secondary metabolites like flavonoids and coumarins shape the root microbiota. Furthermore, secondary metabolite “benzoxazinoids” could act as allelochemicals and natural pesticides on the root microbiome (Hu et al., 2018; Schütz et al., 2019; Jacoby et al., 2020). The symbiotic relationships of plants and endophytic microbes enable them to sustain safely, regardless of extremely harsh environments. The long-term coevolution within ecosystems due to this mutual association, each endophyte evolved a distinct range of hosts, allowing them to colonize a specific host group. The production of secondary metabolites, crucial for endophyte-host communication for mutual survival and their sensitivity to various habitats, is hypothesized to be influenced by the coevolution of endophytes and their host (Lind et al., 2017). Endophytes and their host plants share precursors in their corresponding secondary metabolite in biosynthesis pathways. However, endophytes may mimic the host pathways to establish their own metabolic route for secondary metabolites (Alam et al., 2021). Overall, it has been confirmed that despite their diversity, secondary metabolites are synthesized via a few shared biosynthetic, and the metabolomic pathways of endophytic microbes and their host are similar. Determining whether these secondary metabolites are produced by plants or due to symbiosis with endophytic microorganisms remains disputed. Therefore, understanding the processes influencing plant-microbiome assembly, signaling crosstalk in plant-microbiome communications, genetic controls on secondary metabolites, and how microbiomes and environment alter them are exciting research areas for the future.

## 5 Endophytes-mediated plant-microbe interactions to mitigate environmental cues

**Figure 2:**
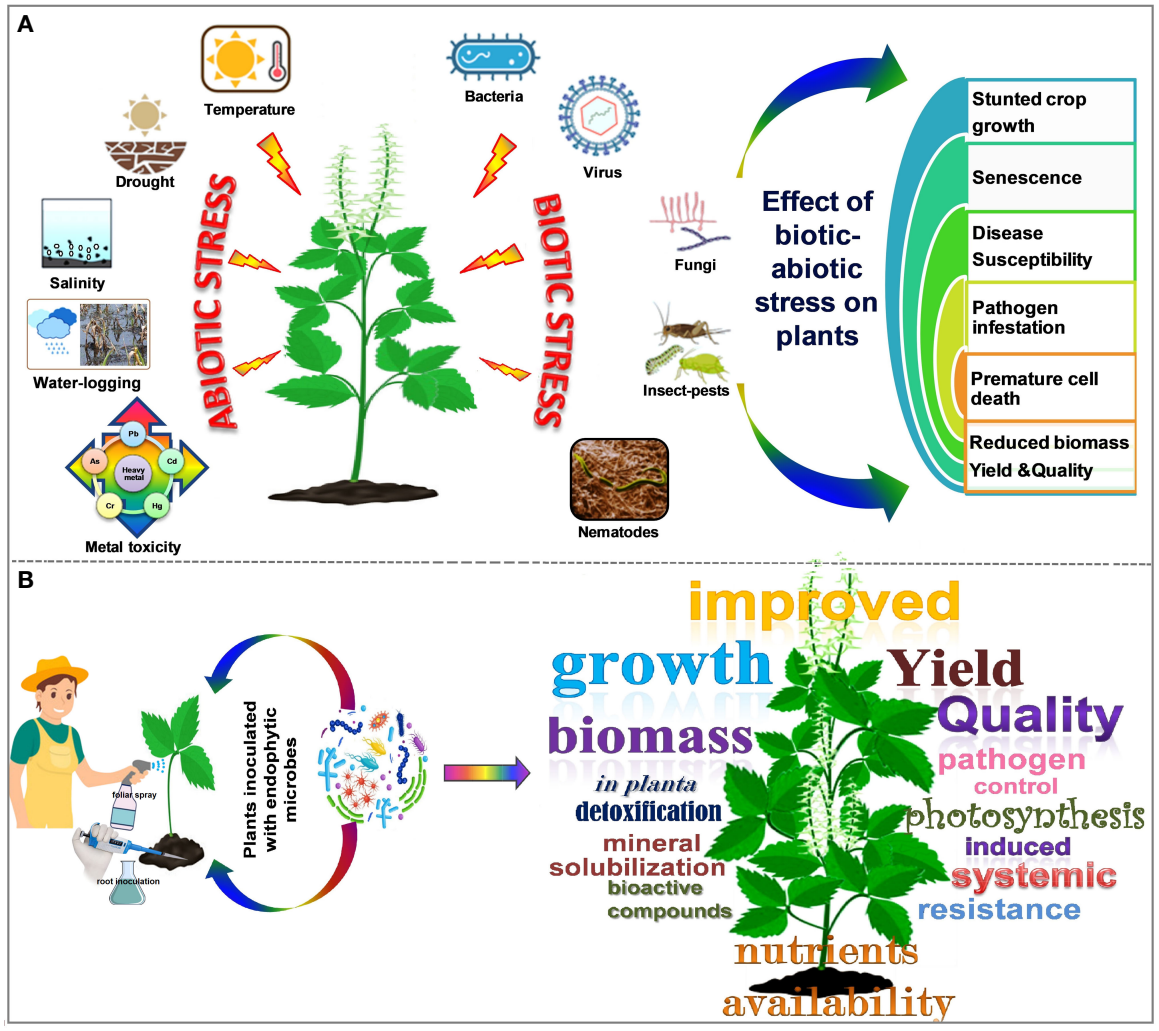
Impact of biotic and abiotic stresses on plants **(A)**, integration of endophytic microbes in plants for improving yield quality and tolerance against different stresses **(B)**.

Plant phenotypic performance is determined by its genotype, environment, and interactions between genotype x environment. The phenotypic potential of a crop is fully expressed in a stress-free environment with no interference from any environmental factors. However, plants endure a range of perturbations categorized into two major groups: (i) weather extremes or abiotic stresses (drought, soil salinity, waterlogging, low and high temperatures, etc.) and (ii) pathogenesis or biotic stresses (bacteria, viruses, fungi, insects, nematodes, etc.). Endophytes improve plants’ stress tolerance by stimulating the synthesis of secondary metabolites (comprising or clinically useful molecules) through various sophisticated strategies (Tripathi A. et al., 2022; Liu et al., 2023). Moreover, they decrease the pressure caused by toxic heavy metals, reduce hazardous greenhouse gases, and limit pests’ growth on plants through a plethora of other specific methods (through extracellular sequestration, modulating antioxidative enzyme activities, mineral nutrient uptake, degradation of pathways for reducing phytotoxicity, etc.) (Azevedo et al., 2000; Stępniewska and Kuźniar, 2013). Remediation by conventional strategies is quite expensive, laborious, and unsustainable, whereas plant-microbe-based approaches for remediation are remarkably potent, less intrusive, and sustainable (Anderson et al., 1993; Radwan, 2009). Additionally, endophytic plants with pertinent metabolic frameworks and degradation pathways toward diminishing phytotoxicity and optimizing decay can rejuvenate groundwater and wastelands (Weyens et al., 2009). Polyaromatic hydrocarbon (PAH) removal by endophytes is also successful; decreasing atmospheric carbon by storing carbon in plants’ rhizospheres is likely a viable strategy (Wu et al., 2009). The schematic representation of the impact of biotic and abiotic perturbations on plants and how the integration of endophytic microbes helps to alleviate these perturbations is illustrated in **Figure 2**.

### 5.1 Endophytic microbes for abiotic stress tolerance in host medicinal plants

Abiotic factors like drought, salt, heat, freezing, heavy metal toxicity, hypoxia and anoxia, waterlogging, and nutritional imbalance are the most severe constraints leading to a drastic decline in crop production (about 51–82%), which hampers global food and nutritional security (Khare and Arora, 2015; Cooke and Leishman, 2016; Yadav et al., 2020; Del Buono, 2021; Raza et al., 2022; Kaur et al., 2023). These stressors have become more common over the past several decades, mainly as a result of the aberrant weather fluctuations triggered by climate change. Plants tolerate these stresses by modifying their physiological, molecular, and biochemical architecture to maintain homeostasis, including osmotic adjustment, nutrient absorption and assimilation, enzyme activity, membrane integrity, metabolic alterations, and most notably, photosynthesis (Moradtalab et al., 2018; Ahanger et al., 2019; Raza, 2021). Most of these imbalances in response to stress conditions are linked to phytohormone synthesis and distribution in plants’ underground and aerial regions (Verma et al., 2016; Arif et al., 2021). Plants generate reactive oxygen species (ROS) as a consequence of these abiotic stresses, which cause severe cell injuries (Oktem et al., 2008; Hasanuzzaman et al., 2020). To counteract the damaging effects of these cues, plants respond physiologically and molecularly, which includes the synthesis of essential proteins associated with metabolism, stimulation of cell signaling, and transcription factors governed through the expression of the majority of stress-tolerant genes that, in turn, are driven by multifaceted biomolecules (Hasanuzzaman et al., 2020; Raza, 2022).

Drought stress has a detrimental effect on plant growth and development, physiological, biochemical, and cellular metabolism, viz., cell membrane elasticity, fluidity, integrity, stomatal conductance, water potential, the structure of enzymes, proteins, amino acids, nucleic acids, etc. and, as well as the homeostasis of the agroecosystems (Kutasy et al., 2022; Noor et al., 2022). Plants modulate diverse cellular signaling pathways, including phytohormones, stress response proteins, osmolytes, and antioxidant enzymes for drought adaptation (Kosar et al., 2021). Numerous endophytes generate ACC deaminase (1-Amino Cyclopropane-1-Carboxylate), which assists its host plant in combating drought by interrupting the ET biosynthesis pathway and diminishing the ET levels, which in turn restricts stress signals. *Bacillus licheniformis* K11, having auxin and ACC deaminase-producing activities, mitigated drought’s detrimental effects without using synthetic agrochemicals (Lim and Kim, 2013). Nevertheless, drought drastically reduces photosynthesis compared to plants’ respiration (Vanlerberghe et al., 2016). Crop plants activate regulons like dehydration-responsive element-binding protein (DREB2) in response to temperature and drought stress (Nakashima et al., 2012). Furthermore, plants produce defensive chemicals in response to drought by mobilizing the metabolites critical for their osmotic adjustment. ABA-mediated stomatal closure may be crucial in controlling plant development by lowering other abiotic stressors, including osmotic stress (Waqas et al., 2012). An endophytic microbe, *Sinorhizobium meliloti* increased FeSOD and CU/ZnSOD, improving drought tolerance in alfalfa (Naya et al., 2007). Likewise, Meng and He (2011) reported an arbuscular mycorrhizal fungus (AMF) maximizes nutrient uptake and modulates metabolic activities (soluble sugar, chlorophyll, leaf subsurface, total phosphorous, total underground nitrogen and tanshinone content, and decreases the content of total aerial nitrogen) to boost drought tolerance in *Salvia*. Moreover, *Trichoderma hamatum* promoted drought tolerance in the Theobroma cacao plant by delaying drought-related stomatal conductance and net photosynthesis adjustments (Bae et al., 2009). Sziderics et al. (2007) claim that a fungus called *Piriformospora indica* increases resistance to osmotic stress by expressing the enzymes ACC-oxidase and lipid transfer protein. The synthesis of ROS under drought conditions often leads to premature cell death (Cruz de Carvalho, 2008), and antioxidant enzymes like catalase (CAT), polyphenol oxidase (PPO), and peroxidase (POD) scavenge ROS to prevent stress-induced damage (Zandalinas et al., 2018). These antioxidants also facilitate rejuvenation from water deficit and dehydration (Laxa et al., 2019). Similarly, *Bacillus amyloliquefaciens* and *Pseudomonas fluorescens* improved drought tolerance in *Mentha piperita* (L.) by enhancing antioxidant enzymes, total phenolic content, and decreasing malondialdehyde (MDA) and proline content (Chiappero et al., 2019). Therefore, antioxidant-producing endophyte microbes are being explored further for favorable eco-friendly gains. Recent research demonstrates the beneficial effects of antioxidant enzymes in peppermint under severe drought (Chiappero et al., 2019; Asghari et al., 2020). Proline accumulation is a key strategy for promoting drought tolerance as it helps in the maintenance of protein structure and function to preserve membrane integrity (Kishor et al., 2005). Besides enhancing antioxidant activity, *Pseudomonas strains* and *Bacillus subtilis* also considerably increased proline levels and total soluble sugars in sweet corn (Zarei et al., 2020). Endophytic microbes have an inherent property to produce phytohormones such as gibberellins (GA), auxin, JA, SA, and ABA. These hormones could also be directly responsible for stimulating various defensive systems in host plants. It has been demonstrated that SA performs an important role in drought stress by altering nitrogen metabolism, inducing the generation of antioxidants, and glycine betaine accumulation, thereby conferring protection from stress (Khan et al., 2022). Shah et al. (2019) reported that *Piriformospora indica* promotes drought tolerance by synthesizing auxins and bioactive compounds in *Cymbidium aloifolium* (L.) Sw. Similarly, *Azospirillum brasilense* and *A. Chroococcum* enhanced drought stress tolerance *via* improving ABA, proteins, phenolic, soluble sugars, flavonoid, and oxygenated monoterpenes while reducing the activity of CAT and GPX in Peppermint (Asghari et al., 2020). An endophyte, *Paenibacillus polymyxa* strain CR1, increased Arabidopsis’s dehydration-responsive genes (RD29), enabling the plants to face drought environments effectively (Liu et al., 2020). Likewise, the GOT9 strain of *Bacillus subtilis* in *Arabidopsis* stimulated the upregulation of several genes related to drought stress, specifically response-to-desiccation (*RD20* and *29B*), encodes dehydrin protein (RAB18), as well as 9-cis epoxy carotenoid dioxygenase (*NCED3*), consequently mitigating the physiological damage caused by drought (Woo et al., 2020). An erratic rainfall pattern due to climate change often functions as an acute stressor, leading to a rapid increase in available soil water, ultimately resulting in premature plant death. Wang et al. (2009) showed that *Penicillium griseofulvin* reduces water stress injury by improving the function of protective enzymes and osmotic levels, thereby increasing the ability to withstand salt, drought, and water stress in *Glycyrrhiza uralensis*. Furthermore, Orchard et al. (2016) claimed that the AMF *Glomus tenue* enhanced the tolerance of ryegrass (*Lolium rigidum*) plants during waterlogging stress. *Pseudomonas putida* inoculation in *Arabidopsis* regulated linked to key polyamine synthetic genes [*ADC* (arginine decarboxylase), *CPA* (N-carbamoyl putrescine amidohydrolase, *AIH* (agmatine iminohydrolase), SPMS (spermine synthase), *SPDS* (spermidine synthase) and *SAMDC* (S-adenosyl methionine decarboxylase)] affecting the amounts of polyamine in cells. The higher level of putrescine and free cellular spermidine is positively linked with water stress (Sen et al., 2018). Recently, *Endostemon obtusifolius* plant inoculated with *Paenibacillus polymyxa* and *Fusarium oxysporum* showed enhanced drought tolerance (Ogbe et al., 2023). In other studies *Streptomyces dioscori* SF1 strain enhanced drought, salinity and phytopathogen resistance in *Glycyrrhiza uralensis* via the production of ammonia, IAA, enzyme activities, potassium solubilization, nitrogen fixation and *Sphingomonas paucimobilis* ZJSH1 strain ameliorate drought, salt, and heavy metal toxicity in *Dendrobium officinale* plants (Li X. et al., 2023; Li J. et al., 2023). Fungal endophytes, *Acrocalymma aquatic* and *Alternaria alstroemeriae* provide tolerance against drought-induced damage in *Isatis indigotica* simply because of synergistic effects on soil enzymatic activity, soil organic material, the biomass of roots, as well as epigoitrin levels (Li W. et al., 2023).

Salinity stress is the most critical abiotic stress that limits crop growth, development, and metabolism, resulting in reduced yield and productivity (Khan et al., 2020 **;** Han et al., 2021). Worldwide, over 6% of the land is classified as saline; this percentage by 2050 is predicted to rise drastically owing to climate change, further aggravating the situation for farming systems. Salinity triggers osmotic pressure, inadequate nutrient supply, and increased ion accumulation beyond critical levels (Hasegawa et al., 2000; Hafeez et al., 2021; Saddiq et al., 2021). Human-generated causes such as irrigation with saline water, industrial pollution, and excessive use of harmful agrochemicals often increase salt stress (Zhu et al., 2019). Different strategies for enhancing plant development under salt stress are triggered by microbial inoculation, including the synthesis of ACC-deaminase, antioxidant enzymes, phytohormones, volatile organic compounds, osmoprotectant metabolites (glycine, proline, alanine, glutamic acid, threonine, serine, choline, betaine, aspartate, and organic acids), modifying ion transporters, which in turn preserves ionic, osmotic, and water homeostasis (Choudhary et al., 2022; Gamalero and Glick, 2022; Kumawat et al., 2022). When sodium ions accumulation reaches toxic levels, ROS is produced that severely damages cellular organelles, viz., mitochondria, chloroplasts, cell membranes, and peroxisomes, impairing plants’ metabolic systems (Munns and Gilliham, 2015). Furthermore, high salinity declined the plant’s water absorption capacity, resulting in poor stomatal activity and reduced cell growth as a consequence of lower cellular water levels. According to Liu et al. (2011), during salt stress, soluble protein content and peroxidase activity (POD) are modulated by endophytic fungi Botrytis *sp.* and *Chaetomium globosum* in Chrysanthemum morifolium. Recently, Jan et al. (2019) claimed salt stress tolerance in Euphorbia milii is promoted by the fungus *Yarrowia lipolytica*. An endophyte, *Brachybacterium paraconglomeratu* strain SMR20, ameliorates salt stress in *Chlorophytum borivilianum* via delaying chlorosis and senescence, enhanced foliar nutrient uptake, deamination of ACC, modifying ET, IAA, ABA, proline, and MDA (Barnawal et al., 2016). Similarly, *Glutamicibacter halophytocola* enhanced tolerance to high NaCl levels in *Limonium sinense* (Qin et al., 2018). de Zélicourt et al. (2018) have demonstrated that an endophyte *Enterobacter* sp. conquers the root and shoot tissues of *Arabidopsis* and promotes salt stress tolerance via producing 2-keto-4-methylthiobutyric. For instance, a bacterial endophyte, *Burkholderia phytofirmans* modified the gene expression for encoding signaling of cell surface component that signals bacteria of environmental stimuli and subsequently enhances their metabolism (Pinedo et al., 2015; Sheibani-Tezerji et al., 2015). Additionally, numerous bacteria in the plant endosphere modify ABA-mediated cell signaling systems as well as their production during salt stress, which may promote plant development. Similarly, *Pseudomonas* PS01 induced salinity tolerance by modulating the expression of stress-responsive genes *LOX2* (lipoxygenase) while reducing *GLY17* (glycogen synthase 17) and *APX2* (ascorbate peroxidase 2*)* in *Arabidopsis* (Chu et al., 2019). A critical factor in managing the nutrient profile and promoting plant growth during salt stress is enhanced microbe-mediated soil enzymatic activity (Shabaan et al., 2022). Recent research revealed that applying *Kosakonia sacchari* to soil can lower antioxidants like CAT, APX, GR (glutathione reductase), and SOD (superoxide dismutase) levels and oxidative stress markers like proline, MDA, and H_2_O_2_ (Shahid et al., 2021). Similarly, *Pseudomonas putida, Klebsiella* sp.*, Alcaligenes* sp., and *P. cedrina* enhanced salt stress tolerance by decreasing the accumulation of MDA, proline, and H_2_O_2_ in *Medicago sativa* (Tirry et al., 2021). Karthikeyan et al. (2012) demonstrated that the inoculation of *Achromobacter xylosoxidans* in *Catharanthus roseus* reduced ET levels and increased the content of antioxidants such as APX, CAT, and SOD under salinity stress. Moreover, halophilic microorganisms control critical stress signaling pathways, such as proline, ABA, and MDA synthesis, ultimately minimizing stress impacts (Ayaz et al., 2022). Likewise, Semwal et al. (2023) reported that *Bacillus* strains NBRI HYL5, NBRIHYL8, and NBRIHYL9 with ACC deaminase activity, biofilm, phosphate solubilization, exo-polysaccharide and alginate generation properties enhanced abiotic stress tolerance in *Gloriosa superb*. Endophytic microbes, *Streptomyces umbrinus* EG1 *and S. carpaticus* EG2 promote root-shoot growth and chlorophyll content, thereby enhancing salt tolerance in *Iris persica and Echium amoenum* plants (Oloumi et al., 2023).

Like drought, salinity, and water stress, global agricultural production is greatly constrained by temperature extremes (heat, cold, and freezing). Heat stress alters the rate of osmotic adjustment, resulting in a disparity in water potential and a negative impact on metabolism and tissue damage. Plants have developed several tolerance mechanisms to cope with such temperature extremes, including the synthesis of heat-shock proteins (HSPs), pathways for eliminating ROS, and the stimulation of certain phytohormones (Khan et al., 2020; Haider et al., 2021; Raza et al., 2021a). The consequences of cold stress, including chilling temperatures of 15°C and freezing temperatures below 0°C, also severely impact the growth and development of plants (Habibi, 2015). Cold-induced abiotic stress profoundly affects all cellular processes in plants, including several signal transduction pathways by which these stressors are transduced, such as ABA, protein kinase, Ca^2+^, protein phosphate, ROS components, etc. The plants’ gene expression is altered in response to surviving cold stress, which modifies osmolytes levels, membrane lipids, phytohormones, proteins, ROS scavenging enzymes, and phenolic content (Ritonga et al., 2021; Saleem et al., 2021; Hwarari et al., 2022; Wei et al., 2022). For example, Fernandez et al. (2012) demonstrated that by balancing carbohydrate metabolism, stress-induced gene expression, and increased metabolite levels, *Burkholderia phytofirmans* PsJN bacterized grapevine showed enhanced tolerance against low temperature. Similarly, Su et al. (2015)) discovered that treating *Arabidopsis thaliana* with *Burkholderia phytofirmans* PsJN during cold stress curtailed the plasmalemmas’ disruption and strengthened the mesophyll cell wall. In other studies, PsJN ameliorated cold tolerance in *Vitis vinifera* with an improved accumulation of proline, aldehydes (ALD), and MDA along with *PAL* (phenylalanine ammonia-lyase) and *STS* (stilbene synthase) genes (Theocharis et al., 2012) as well as improved CO2 fixation, starch and phenolics (Barka et al., 2006). However, the *Dichanthelium lanuginosum* plant relies on endophytic fungi Curvularia protuberate *in three-way mutualistic interactions with a virus (virus-fungal endophyte-plant)* for survival at high soil temperatures (Márquez et al., 2007).

Metal toxicity is increasing globally due to anthropogenic activities that have not only polluted the soil but also pose a severe threat to human health when they reach the food chain and are biomagnified. Heavy metals like arsenic (As), cadmium (Cd), lead (Pb), mercury (Hg), aluminum (Al), copper (Cu), and zinc (Zn) supplied through irrigation significantly influenced soil dynamics (Nazli et al., 2020; Mehmood et al., 2019; Bashir et al., 2021; Haseeb et al., 2022). The deleterious effects of heavy metal ions on tissues, such as the stimulation of necrosis and chlorosis, inhibition of chlorophyll biosynthesis, and membrane lipid degradation, may significantly impact crop productivity (Takasaki et al., 2010; Raza et al., 2021b; Raza et al., 2022). Plants have evolved sophisticated mechanisms, including hyperaccumulation, tolerance, exclusion, and chelation with organic compounds as the fundamental strategies. Research findings have suggested that endophytic microorganisms play a significant role in boosting resilience to metal toxicity via complex mechanisms, including intracellular accumulation, sequestration, extracellular precipitation, and conversion of toxic metals to a negligible or non-toxic form (Rajkumar et al., 2009; Ma et al., 2016; Mishra et al., 2017). Interestingly, Domka et al. (2019) discovered a fungal endophyte called *Mucor* sp. significantly strengthens the ability of *Arabidopsis arinosa* to tolerate metal toxicity. Furthermore, an endophyte, *Bacillus* sp. *SLS18* diminishes the toxicity of heavy metals by accumulating biomass in the root tillers and leaves of *Solanum nigrum* and *Phytolacca acinosa* (Luo et al., 2012). Similarly, microbial endophytes *Paenibacillus hunanensis* strain CIMAP-A4 and BAC-7 improved arsenic tolerance in *Bacopa monnieri* (L.) via IAA production and biofilm formation (Tripathi P. et al., 2022). Xu et al. (2016) claimed that *Agrobacterium* spp. *and Bacillus* spp. reduced arsenate to arsenite in *Pteris vittata* (L.). An endophyte, *Paenibacillus* relieved heavy metal toxicity in *Tridax procumbens* (Govarthanan et al., 2016) as well as helped in the removal of PAHs phytotoxicity via biodegradation of phenanthrene through co-metabolism in *Plantago asiatica* (Zhu et al., 2016). Endophytic microorganisms can also diminish heavy metal-induced oxidative-stress damage (Wan et al., 2012). The toxic effects of Cd accumulation were synergistically controlled by various plant metabolic defensive systems, including hyperaccumulators, detoxification routes, and antioxidative processes by bacterial endophytes, *Klenkia*, *Modestobacter, Sphingomonas* in *Lonicera japonica* (Xie et al., 2023) *Pseudomonas* strain E3 in *Solanum nigrum* (Chi et al., 2023).

### 5.2 Endophytic microbes for biotic stress tolerance in host medicinal plants

Biotic stresses are known to be affected by abiotic stress conditions in terms of their incidence and dissemination (Scherm and Coakley, 2003; McDonald et al., 2009; Ziska et al., 2010; Peters et al., 2014). Through modifications to plant physiology and defense mechanisms, these stress conditions also directly impact plant-pest interactions (Scherm and Coakley, 2003; Duveiller et al., 2007; Gimenez et al., 2018). Several biological agents, including bacteria, fungi, viruses, weeds, insects, and nematodes, are the major stress factors that tend to increase ROS, affecting how well plants operate physiologically and molecularly and decreasing agricultural productivity. Plant-parasitic nematodes can attack all parts of the plant, although they predominantly harm the root system and spread disease through the soil. They cause stunting and wilting, which are symptoms of inadequate nutrition. Although they seldom kill, their hosts’ viruses can harm plants systemically, producing stunting, chlorosis, and malformations in different regions of the plant. Piercing-sucking insects can spread viruses to plants via their styles. In combination with bacteria, fungi cause a more severe impact, resulting in vascular wilts, leaf spots, and cankers (Schlenker and Roberts, 2009). Insects may physically harm plants severely, including the leaves, stems, bark, and flowers, while infected plants can transmit viruses and bacteria to healthy plants via insects.

In many cases, weeds can take over habitats faster than certain attractive plants because they proliferate and generate many viable seeds. Inhibiting the growth of desirable plants, such as crops or flowers, is not done directly by weeds, which are viewed as undesired and unproductive plants, but rather through competing with the desirable plants for nutrients and space. Through antagonistic action, endophytic microbes can strengthen plants’ defense systems against pathogen invasion (Miller et al., 2002; Gunatilaka, 2006). Additionally, they are said to improve the health of the soil and crops by assisting plants in coping with biotic stress. Therefore, using endophytic microbes as biofertilizers and biocontrol agents has established a natural alternative to harmful chemicals for crop production and alleviating biotic stress. In general, two mechanisms, systemic-acquired resistance (SAR) and induced systemic resistance (ISR), confer plant resistance to pathogens. ISR is defined as the plants’ innate resistance primarily mediated by beneficial microbes via modulating root immunity, root colonization, and the production of specific elicitors like volatile organic compounds, siderophores, polysaccharides, enzymes, and phytohormones, whereas SAR is considered as the plants’ acquired resistance (Olowe et al., 2020; Hamid et al., 2021).

A wide range of pests and pathogens can be successfully combated using the SAR and ISR mechanisms (Vlot et al., 2021; Meena et al., 2022; Yu et al., 2022). Even though multiple studies have shown that endophytic microbes regulate diversified physiological, cellular, and molecular functions in plants and aid in their survival when attacked by pathogens (Teixeira et al., 2019; Olowe et al., 2020; Castiglione et al., 2021; Yu et al., 2022), unfortunately, the fundamental mode of action of pathogenesis has yet to be discovered. The results of comprehensive investigations show that developing resistance to several pathogens, such as bacteria, viruses, and fungi, relies on complex mechanisms that may operate simultaneously (Yu et al., 2022), including stimulation of several defense response genes and enzymes (CAT, GPX (guaiacol peroxidase), APX, GR, SOD, and POD), accumulation of hormones (auxin, GA, ET, JA, and SA), glucanases, sugars, chitinases, PR proteins, secondary metabolites and osmolytes which in turn play a direct role in limiting the growth and spread of pathogens (Baxter et al., 2014; Pieterse et al., 2014; Conrath et al., 2015; Camejo et al., 2016; Guo et al., 2019; Olowe et al., 2020; Luo et al., 2022). Previous research confirmed that endophytes significantly control the host’s gene expression, physiological responses, and defense-related processes in plants (Van Bael et al., 2012; Estrada et al., 2013; Salam et al., 2017). For instance, JA and SA prove to be very helpful in plant stress responses against phytopathogens (Mejía et al., 2008; Ren and Dai, 2012; Khare et al., 2016). Furthermore, the gibberellins synthesized by endophytes boost insect and pathogens’ resistance via SA and JA pathways (Waqas et al., 2015a). *Fusarium solani*, an endophyte, induces systemic resistance to the pathogenic fungi *Septoria lycopersici* by promoting the expression of genes associated with the pathogenesis (Kavroulakis et al., 2007). Additionally, some endophytic microbes produce an array of bioactive compounds that might improve the plants’ resistance to different phytopathogens such as *Macrophomina phaseolina*, which causes charcoal rot disease via siderophores-synthesizing (Arora et al., 2001), *Vertcillium* wilt (Mercado-Blanco et al., 2004), *Cadosporium sphaerospermum and C. cladosporioides* through the synthesis of pathogen-toxic cadinane sesquiterpenoids (Silva et al., 2006), antagonistic to pathogenic fungi by toxic chemical “trichothecin” (Zhang et al., 2010), *Fusarium oxysporum* and *F. Solani* (Yang et al., 2012), *Rhizoctonia solani, Pythium myriotylum, Phytophthora capsici, Colletotrichum gloeosporioides*, and *Radopholus similis* by producing volatile substances(Sheoran et al., 2015) as well as inhibiting pathogenic fungi by releasing some toxins (Wang et al. (2012). According to Strobel et al. (1999), an endophytic microbe *Cryptosporiopsis cf. quercina* in *Triptergyium wilfordii* (thunder god vine) produces “cryptocin” and “cryptocandin,” which are poisonous to the host plant’s pathogenic fungus *Pyricularia oryzae*. Moreover, Cao et al. (2009) reported endophytes, *Stachybotrys elegans, Choiromyces aboriginum, and Cylindrocarpon* linked with cell wall-disruptive enzymes combat pathogenic fungi in *Phragmites australis* plant. Microbial endophytes viz., *Cohnella* sp.*, Paenibacillus* sp., and *Pantoea* sp. induced plant defense mechanism against anthracnose disease in *Centella asiatica* (Rakotoniriana et al., 2013). In other studies, *Bacillus amyloliquefaciens* improved tolerance to root-rot in *Panax notoginseng* (Ma et al., 2013), phytophthora blight resistance in *Ginkgo biloba* (Yang et al., 2014), and inhibited multiple phytopathogens in *Curcuma longa* via synthesizing ‘iturin’ and ‘surfactin’ (Jayakumar et al., 2019). Hong et al. (2018) reported that microbial endophytes, *Stenotrophomonas maltophilia* and *Bacillus* sp. suppressed phytopathogens growth in *Panax ginseng.* Fungal endophytes, *Penicillium chrysogenum* and *Alternaria alternate* enhanced tolerance against pathogenic microorganisms in *Asclepias sinaica* by producing extracellular enzymes viz., amylase, pectinase, xylanase, cellulase, gelatinase, and tyrosinase (Fouda et al., 2015). In another study, *Withania somnifera* plants inoculated with *Talaromyces trachyspermus* effectively combat phytopathogens which resulted from the antagonistic activity of endophytes and enhanced IAA, phosphate solubilization, and siderophore synthesis (Sahu et al., 2019). Similarly, Jiang et al. (2018) showed that *Bacillus velezensis* increased plants’ resistance to gray mold disease caused by *Botrytis cinerea* by activating antioxidant-mediated defense signaling genes SOD, POD, CAT, and SA-signaling genes viz., NPR1 (non-expressor of pathogenesis-related genes) and PR1 (pathogenesis-related protein1). These findings suggest that endophyte priming triggers molecular and biochemical changes that prevent pathogen invasions of plants. Interestingly, Kumar et al. (2016) identified that the inoculation of the endophyte *Peanibacillus lentimorbus* in *Nicotiana tabacum* reduced the prevalence of CMV (cucumber mosaic virus) by augmenting the expression of genes related to stress *PR1*, *AsSyn* (asparagine synthetase), *Gluc* (b-1,3-glucanase), *BR-SK1*(brassinosteroid signaling kinase 1), *TCAS* (tetra-hydrocannabinolic acid synthase), *ZF-HD* (zinc finger-homeodomain), *RdRP2* (RNA dependent RNA polymerase), and antioxidants (CAT, SOD, APX, and GPX). Recently, Azabou et al. (2020) reported that an endophyte *Bacillus velezensis* OEE1 prevents *Verticillium* wilt disease in olive plants by producing antifungal volatile organic molecules (benzene acetic acid, 1-decene, phenyl ethyl alcohol, tetradecane, and benzaldehyde). Likewise, *Microbacterium* sp. SMR1 enhanced downy mildew tolerance in *Papaver somniferum* (L.) via protein modification, differential expression of transcripts related to signal transduction, transcription factors, and SA-dependent defense pathway (Ray et al., 2021). Many researchers showed the ability of both bacterial and fungal endophytes to control diseases and phytopathogens by synthesized volatile and non-volatile compounds, soluble antifungal metabolites and by specific mechanisms including activation of defense enzymes and PR proteins associated with ISR, JA/ET mediated disease resistance, antagonism, antimicrobial, antioxidant, and anti-proliferative properties, production of IAA, siderophores.and β-1,3-glucanase, proteolytic activity, chitinase and cellulose synthesis in diverse medicinal plants including *Chloranthus elatior*, *Taxillus chinensis, Salvia miltiorrhiza, Curcuma longa, Dioscorea bulbifera, Viola odorata, Cremastra appendiculata*, *Angelica sinensis*, *Cornus florida, Nicotiana tabacum, Zingiber zerumbet and Piper betle* (Harsha et al., 2023; Jiao et al., 2023; Manasa et al., 2023; Mei et al., 2023; Rotich and Mmbaga, 2023; Salwan et al., 2023; Santra and Banerjee, 2023; Sharma et al., 2023; Song et al., 2023; Thankam and Manuel, 2023; Wang et al., 2023; Yehia, 2023; Zou et al., 2023).

It is well documented that endophytic microbes improve host plant resistance to insect herbivores primarily by synthesizing a variety of alkaloid-based protective chemicals in the plant tissue or by changing the nutritional quality of the plant. Eventually, endophytes such as *Chaetomium cochliodes, Trichoderma viride*, and *Cladosporium cladosporioide* are known to facilitate insect resistance in creeping thistle (Gange et al., 2012) and red spruce (Sumarah et al., 2010). An endophyte, *Epichloë coenophiala* AR584, showed enhanced herbivore resistance in *Lolium arundinaceum* (Schreb.) *via* the production of alkaloids which provide anti-herbivore defenses, stoichiometry, photosynthesis, and transpiration rates, and stomatal conductance (Johnson et al., 2023). Endophytes function as an acquired plant immune system, taking up space, fighting diseases that may otherwise attack the host, and delaying or deterring herbivores’ infection. For instance, Bittleston et al. (2011) showed that an endophyte *Leucocoprinus gongylophorus* produces compounds that are antagonistic fungal-ants’ symbionts to boost insect resistance. Furthermore, an endophyte *Chaetomium Ch1001* increases resistance to the root-knot nematode by synthesizing ABA that affects the insect juveniles’ second-stage motility (Yan et al., 2011). Additionally, endophytes *Beauveria bassiana* and *Lecanicillium dimorphum* improve insect resistance by altering cell division-related protein expressions in the host plant (Gómez-Vidal et al., 2009). Daungfu et al. (2019) found that bacterial endophytes *Bacillus subtilis* LE24, B. amyloliquefaciens LE109, and B. tequilensis PO80 from the citrus plant with antagonistic properties against phytopathogens might be helpful in the biocontrol of diseases. Diab et al. (2023) recently claimed that endophytic microbes, *Streptomyces* sp. *ES2, Streptomyces, Nocardioides*, and *Pseudonocardia* produce metabolites that act as natural biocontrol agents against insects in *Artemisia herba-alba* and *A. judaica* plants. A list of endophytic microbes enhancing abiotic and biotic stress tolerance and associated mechanisms in the host plants are shown in **Table 1** (bacterial endophytes) and **Table 2** (fungal endophytes).

**Table 1:** Biotic-abiotic stress tolerance and plant defense mechanism conferred by endophytic bacteria in host medicinal plants.

Endophytic microbes	Host medicinal plants	Stress type	Plant defense mechanism	References
*Sinorhizobium meliloti*	*Medicago sativa* (L.)	Drought	FeSOD and CU/ZnSOD are up-regulated	Naya et al., 2007
*Bacillus amyloliquefaciens, Pseudomonas fluorescens*	*Mentha piperita* (L.)	Drought	Enhance antioxidant enzymes (POX and SOD), total phenolic content, decrease MDA and proline	Chiappero et al., 2019
*Azospirillum brasilense, Azotobacter chroococcum*	*Mentha piperita* (L.)	Drought	Improve ABA, proteins, SOD, phenolic, soluble sugars, flavonoid, and oxygenated monoterpenes, while reducing the activity of CAT and GPX	Asghari et al., 2020
*Fusarium oxysporum* (EOLF-5)	*Endostemon obtusifolius* (E. Mey. ex Benth.) NE Br.	Drought	Production of ammonia and siderophore, free radical scavenging ability	Ogbe et al., 2023
*Acrocalymma aquatica Alternaria alstroemeriae*	*Isatis indigotica* Fortune	Drought	Via synergistic effects on soil enzymatic activities, organic matter, root biomass, epigoitrin content	Li W. et al., 2023
*Pseudomonas putida, Klebsiella* sp.*, Alcaligenes* sp.*, P. cedrina*	*Medicago sativa* (L.)	Salinity	Decrease accumulation of MDA, proline and H_2_O_2_	Tirry et al., 2021
*Enterobacter* sp. *SA187*	*Citrus* (L.)	Salinity	Ethylene stimulation	de Zélicourt et al., 2018
*Burkholderia phytofirmans*	*Arabidopsis* *Thaliana* (L.) Heynh.	Salinity	Improve proline and modulate genes responsible for ABA signaling (RD29, RD29B), antioxidant linked(APX2), glyoxylate pathway (GYLI7), reduce expression of JA signaling gene (LOX2)	Pinedo et al., 2015
*Bacillus megaterium*	*Arabidopsis* *Thaliana* (L.) Heynh.	Salinity	Enhanced CYP94B3 (linked with JA-Ile catabolism)	Erice et al., 2017
*Bacillus amyloliquefaciens*	*Arabidopsis* *Thaliana* (L.) Heynh.	Salinity	Up-regulation of genes responsible for antioxidant (POX and GST), ET-signaling (ACS7, ACS11, ACS2, and ACS8), JA-signaling (LOX), down-regulating ABA-signaling (NCED3, ABI1, NCED4, and MARD1)	Liu et al., 2017
*Brachybacterium paraconglomeratu strain* SMR20	*Chlorophytum borivilianum Santapau* & R.R.Fern.	Salinity	Deamination of ACC, delayed chlorosis and senescence, reducing stress ethylene, modifying IAA and ABA levels, alteration of leaf pigments, proline, malondialdehyde, and enhanced foliar nutrient uptake	Barnawal et al., 2016
*Achromobacter xylosoxidans*	*Catharanthus roseus* (L.) G. Don	Salinity	Increased germination percentage and root weight under saline conditions	Karthikeyan et al., 2012
*Glutamicibacter halophytocola*	*Limonium sinense* (Girard) Kuntze	Salinity	Improved tolerance to high NaCl concentration	Qin et al., 2018
*Streptomyces umbrinus* EG1 and *Streptomyces carpaticus* EG2	*Iris persica* L. and *Echium amoenum* Fisch. & C.A.Mey.	Salinity	Promotes root and shoot growth and chlorophyll content	Oloumi et al., 2023
*Bacillus*, *Brevibacillus*, *Agrobacterium*, and *Paenibacillus*	*Vicia faba* L.	Salinity	By decreasing growth parameters and metabolic activities, and increasingcproline content and of antioxidant enzymes activity	Mahgoub et al., 2021
*Bacillus subtilis,B. tequilensis, B. licheniformis, B. sonorensis Burkholderia* sp.*, Acinetobacter pittii*	*Artemisia annua* (L.)	Water, drought, and salinity	Improving artemisinin yield and content by siderophore production, phosphate solubilization, IAA production, ACC deaminase activity and nitrogen fixation	Tripathi et al., 2020
*Bacillus* sp. *strain* NBRI HYL5, NBRIHYL8, NBRIHYL9	*Gloriosa superba* L.	Abiotic stress	ACC deaminase activity, biofilm, phosphate solubilization, IAA, exo-polysaccharide and alginate generation	Semwal et al., 2023
*Burkholderia phytofirmans strain* PsJN	*Vitis vinifera* (L.)	Chilling	Enhancement of chilling tolerance	Barka et al., 2006
*Burkholderia phytofirmans (PsJN)*	*Vitis vinifera* (L.)	Cold	Balancing carbohydrate metabolism	Fernandez et al., 2012
*Bacillus* sp. SLS18	*Solanum nigrum* (L.)*, Phytolacca acinosa* Roxb.	Heavy metal toxicity (Mn and Cd)	Improving biomass and root tillers accumulation	Luo et al., 2012
*Pseudomonas koreensis* AGB-1	*Miscanthus sinensis* Andersson	Heavy metal toxicity (Zn Cd As and Pb)	Through extracellular sequestration, increased Catalase and SOD activities	Babu et al., 2015
*Serratia nematodiphila* LRE07	*Solanum nigrum* (L.)	Heavy metal promoted oxidative injury	Improving essential mineral nutrient uptake and antioxidative enzymes activities	Wan et al., 2012
*Paenibacillus hunanensis* strain CIMAP-A4, BAC-7	*Bacopa monnieri* (L.)	Heavy metal toxicity (Arsenic)	IAA production and biofilm formation	Tripathi P. et al., 2022
*Bacillus gaemokensis* strain CIMAP-A7	*Andrographis paniculata* (Burm.f.) Nees	Phytotoxicity (Atrazine)	By reducing stress enzymes, proline, and malondialdehyde accumulation	Tripathi et al., 2021
*Paenibacillus* sp.	*Tridax procumbens* (L.)	Heavy metal toxicity	Relieved heavy metal stress in plants	Govarthanan et al., 2016
*Agrobacterium* spp. *and Bacillus* spp.	*Pteris vittata* (L.)	Heavy metal toxicity (Arsenic)	Reduced arsenate to arsenite	Xu et al., 2016
*Paenibacillus* sp.	*Plantago asiatica* (L.)	Phytotoxicity Polycyclic aromatic hydrocarbons (PAHs)	Biodegradation of phenanthrene through co-metabolism	Zhu et al., 2016
*Klenkia*, *Modestobacter, Sphingomonas*	*Lonicera japonica* thunb	Heavy metal-toxicity	The toxic effects of Cd accumulation were synergistically controlled by various plant metabolic defensive systems viz., detoxification routes and antioxidative processes	Xie et al., 2023
*Pseudomonas strain* E3	*Solanum nigrum* L.	Heavy metal-toxicity	By increasing cadmium (Cd) extraction via hyperaccumulator	Chi et al., 2023
*Pseudomonas fluorescence*	*Olea europaea* (L.)	Disease	Antagonism	Mercado-Blanco et al., 2004
*Penicillium citrinum* LWL4, *Aspergillus terreus* LWL5	*Helianthus annuus* (L.)	Disease	Modulation of antioxidants, defense hormones, and functional amino acids	Waqas et al., 2015b
*Bacillus* *amyloliquefaciens*	*Nicotiana tobaccum* (L.)	Disease	Regulate expression of PPO, JA/ET signaling	Jiao et al., 2020
*Microbacterium* sp. SMR1	*Papaver somniferum* (L.)	Disease (Downy mildew)	By protein modification, differential expression of transcripts related to signal transduction, transcription factors, SA-dependent defense pathway	Ray et al., 2021
*Bacillus amyloliquefaciens*	*Panax notoginseng* (Burkill) F.H. Chen.	Disease (Root-rot)	Antagonistism	Ma et al., 2013
*Cohnella* sp.*, Paenibacillus* sp. *and Pantoea* sp.	*Centella asiatica* (L.) Urban	Disease (Anthracnose)	Induction of plant defense mechanism, antagonism	Rakotoniriana et al., 2013
*Bacillus amyloliquefaciens*	*Ginkgo biloba* (L.)	Disease (*Phytophthora* blight)	Produced antibiotics and induced systemic resistance	Yang et al., 2014
*Bacillus* sp.	*Curcuma longa* (L.)	Disease	Induced host disease resistance	Jayakumar et al., 2019
*Stenotrophomonas* sp.*, Serratia marcescens, Bacillus thuringiensis*	*Cornus florida* L.	Disease	Activation of defense enzymes and PR proteins associated with induced systemic resistance	Rotich and Mmbaga, 2023
*Bacillus amyloliquefaciens*	*Nicotiana tabacum* L.	Disease	By activation of JA/ET mediated disease resistance	Jiao et al., 2023
*Bacillus* spp.*, Klebsiella aerogenes, Pseudomonas fuscovaginae, Enterobacter tabaci, Pantoea* spp.*, Kosakonia* spp.	Zingiber zerumbet (L) Smith	Disease	Antagonism, biocontrol agents for soil-borne soft-rot disease (*Pythium* spp.)	Harsha et al., 2023
*Bacillus velezensis*	*Piper betle* L.	Disease	Through induction of defense enzymes	Manasa et al., 2023
*Peanibacillus lentimorbus B-30488*	*Nicotiana tobaccum* (L.).	Virus	Targets antioxidant enzymes and PR genes	Kumar et al., 2016
*Streptomyces* sp. *ES2, Streptomyces, Nocardioides, and Pseudonocardia*	*Artemisia herba-alba* Asso, *A. judaica* L.	Insect	By producing metabolites that acts as natural biocontrol agents	Diab et al., 2023
*Bacillus subtilis, Myxormia* sp.	*Angelica sinensis* (Oliv.) Diels	Pathogenic fungi	Secretes some toxic chemicals harmful to pathogens *viz., Fusarium oxysporum*, *F. Solani*	Yang et al., 2012
*Bacillus subtilis LE24, B. amyloliquefaciens LE109, B. tequilensis PO80*	*Citrus* (L.)	Phytopathogen	Pathogen biocontrol	Daungfu et al., 2019
*Pseudomonas putida BP25*	*Piper nigrum* (L.)	Phytopathogen	Suppression of pathogens	Sheoran et al., 2015
*Bacillus velezensis* OEE1	*Olea europaea* (L.)	Pathogenic fungi: *Verticillium* *dahliae*	Producing antifungal lipopeptides and secondary metabolites	Azabou et al., 2020
*Phyllobacterium myrsinacearum*	*Epimedium brevicornu* Maxim	Phytopathogenes	Antagonistism	He et al., 2009
*Stenotrophomonas maltophilia and Bacillus* sp.	*Panax ginseng* C.A. Meyer	Phytopathogenic fungi	Suppressed pathogen growth	Hong et al., 2018
*Pantoea*, *Agrobacterium*, *Pseudomonas*, *Bacillus* sp., *Colletotrichum* sp., *Trichothecium roseum*, *Phomopsis liquidambari*	*Artemisia annua* L.	Phytopathogens	Antagonistic activity	Zheng et al., 2021
*Pseudomonas* sp. SWUSTb-19	*Aconitum carmichaelii* Debx	Pathogenic fungi	Antagonism, bio-control agent against southern blight	Zou et al., 2023
*Bacillus amyloliquefaciens* SNMB1	*Salvia miltiorrhiza* Bunge	Phytopathogens and salinity	Antifungal activity	Mei et al., 2023
*Kocuria rocea, Bacillus subtilis, Brevibacterium casei, Actinobacterium JS14 strain, B. Amyloliquefaciens, B. velezensis*	*Curcuma longa* L.	Phytopathogens and salinity	Antimicrobial properties, producing hormones viz., IAA, GA, CT and secondary metabolites	Thankam and Manuel, 2023
*Clonostachys pseudochroleucha*, *Parathyridaria percutanea, Curvularia lunata*	*Dioscorea bulbifera* L.	Phytopathogens	Phosphate solubilisation, siderophore, IAA, and HCN production, amylase, lipolytic, protease, cellulolytic and chitinase activity	Sharma et al., 2023

**Table 2:** Biotic-abiotic stress tolerance and plant defense mechanism conferred by endophytic fungi in host medicinal plants.

Endophytic microbes	Host medicinal plants	Stress type	Plant defense mechanism	References
*Piriformospora indica*	*Cymbidium aloifolium* (L.) Sw.	Drought and pathogen	By synthesizing auxins and bioactive compounds	Shah et al., 2019
*Trichoderma hamatum* DIS 219b	*Theobroma cacao* (L.)	Drought	Drought-induced adaptation in stomatal closure and net photosynthesis	Bae et al., 2009
*Paenibacillus polymyxa* (EORB-2)	*Endostemon obtusifolius* (E. Mey. ex Benth.) N.E. Br.	Drought	Production of ammonia and siderophore, free radical scavenging ability	Ogbe et al., 2023
*Streptomyces dioscori* SF1	*Glycyrrhiza uralensis* Fisch. ex DC.	Drought, salinity, phytopathogens	Via production of ammonia, IAA, enzymes activities, potassium solubilization, nitrogen fixation	Li X. et al., 2023
*Sphingomonas paucimobilis* ZJSH1	*Dendrobium officinale* Kimura et. Migo	Drought, salt, and heavy metal toxicity	By hormones (IAA, SA, ABA and zeaxanthin), phosphate cycle, antioxidant enzymes, and polysaccharides	Li J. et al., 2023
*Funneliformis mosseae, Rhizophagus intraradices, Claroideoglomus etunicatum*	*Sesbania sesban* (L.) Merr.	Salinity	Secrets phytohormones	Abd Allah et al., 2015
*Yarrowia lipolytica*	*Euphorbia milii* Des Moul.	Salinity	By producing IAA, IAM (indole-3-acetamide), phenol, and flavonoid	Jan et al., 2019
*Chaetomium globosum, Botrytis* sp.	*Chrysanthemum morifolium* (Ramat.) Hemsl.	Salinity	Increase POD activity and soluble protein content	Liu et al., 2011
*Glomus mosseae*, *G. microcarpum*, *G. fasciculatum*, *G.intraradices*, *Gigaspora margarita*, and *Gigaspora heterogama*	*Jatropha curcas* (L.)	Salinity	By improving physiological parameters (leaf relative water content, chlorophyll, proline, and soluble sugar), antioxidant enzymes (SOD, POD, APX, GR), and by reducing oxidative damage to lipids	Kumar et al., 2015
*Arbuseular mycorrhiza, Penicillium griseofulvum*	*Glycyrrhiza uralensis* Fisch. ex DC.	Water, drought, and salinity	Improving the activity of protective enzymes and osmotic levels	Wang et al., 2009
*Glomus tenue*	*Lolium rigidum* Gaud.	Waterlogging	By improving root length and other morpho-physiological mechanisms	Orchard et al., 2016
*Piriformospora indica*	*Capsicum annum* (L.)	Osmotic stress	Encoding lipid transfer protein and ACC-oxidase enzyme	Sziderics et al., 2007
*Curvularia protuberate*	*Dichanthelium lanuginosum* (Ell.) Gould	Heat	Mutualism	Márquez et al., 2007
*Mucor* sp.	*Arabidopsis arenosa* (L.) Lawalrée	Heavy metal-induced oxidative stress	Down-regulating catalase activity	Domka et al., 2019
*Preussia africana, Bjerkandera adusta, Schizophyllum commune, Alternaria embellisia, Trichaptum biforme, Septoria malagutii, A. consortiale, Verticillium dahliae, Fusarium avenacearum, Trametes versicolor*	*Anthemis altissima* L.*, Matricaria parthenium* L.*, Cichorium intybus* L., *Achillea millefolium* L.*, A. filipendulina* Lam.	Abiotic stress	Produced the highest level of IAA-like compounds which enhances seed germination	Hatamzadeh et al., 2023
*Epulorhiza* sp.	*Anoectochillus formosanus* Hayata	Abiotic stress	Strengthen enzyme activities which enhances survival rate of seedlings	Tang et al., 2008
*Sclerotium* sp.	*Atracty lancea* (Thunb.) DC.	Abiotic stress	Improving the protection of cells from desiccation and metabolism of the host, enhancing survival rate of seedlings	Chen et al., 2008
*Colletotrichum tropicale*	*Theobroma cacao* (L.)	Disease (frosty pod rot, witches broom, black pod rot)	Antagonism	Mejía et al., 2008
*Epulorhiza* sp. AR-18	*Anoectochilus roxburghii* (wall.) Lindl	Disease	Production of siderophore	Arora et al., 2001
*Colleto trichum gloeosporioides*, *Trichoderma tomentosum, Colletotrichum godetiae, Talaromyces amestolkiae*	*Cremastra appendiculata* (D.Don) Makino	Disease	Antagonism, production of IAA, siderophores.and β-1,3-glucanase, proteolytic activity, chitinase and cellulose synthesis	Wang et al., 2023
*Colletotrichum acutatum*	*Angelica sinensis* (Oliv.) Diels	Disease	Antimicrobial, antioxidant, and anti-proliferative properties	Yehia, 2023
*Leucocoprinus gongylophorus*	*Cordia alliodora* Cham.	Insect	Release some toxins, antagonism	Bittleston et al., 2011
*Chaetomium cochliodes, Cladosporium cladosporioides, Trichoderma viride*	*Cirsium arvense* (L.) Scop.	Insect	Release some toxic chemicals harmful to pathogens	Gange et al., 2012
*Beauveria bassiana, Lecanicillium dimorphum, L*. cf. *Psalliotae*	*Phoenix dactylifera* (L.)	Insect	Regulate cell division-related proteins expression in the host	Gómez-Vidal et al., 2009
*Penicillium citrinum LWL4, Aspergillus terreus LWL5*	*Helianthus annuus (L.)*	Insect	Salicylic and jasmonic acid pathways	Waqas et al., 2015a
*Penicillium rubens* (150 strains)	*Picea glauca* (Moench) Voss	Insect	Release toxic chemicals	Sumarah et al., 2010
*Epichloë coenophiala* AR584	*Lolium arundinaceum* (Schreb.)	Biotic (Herbivore attack)	Stoichiometry, secretion of certain alkaloids which provide anti-herbivore defences	Johnson et al., 2023
*Paraphaeosphaeria* sp.	*Vaccinium myrtillus*	Pathogenic fungi	Flavonoid biosynthesis and degradation	Koskimäki et al., 2009
*Choiromyces aboriginum, Stachybotrys elegans, Cylindrocarpon*	*Phragmites australis* (Cav.) Steud.	Pathogenic fungi	Produce cell wall-degrading enzymes to kill pathogenic fungi	Cao et al., 2009
*Gilmaniella* sp. AL12.	*Atractylodes lancea* (Thunb.) DC.	Pathogenic fungi	Production of JA-inducing defense responses	Ren and Dai, 2012
*Chaetomium globosum* L18	*Curcuma wenyujin* Y.H.Chen & C.Ling	Pathogenic fungi	Produce some toxic chemicals harmful to pathogens	Wang et al., 2012
*Trichothecium roseum*	*Maytenus hookeri* Loes.	Pathogenic fungi	Release “trichothecin” toxic to phytopathogens	Zhang et al., 2010
*Phomopsis cassia*	*Cassia spectabilis* DC.	Pathogenic fungi	Produce cadinane sesquiterpenoids toxic to pathogens	Silva et al., 2006
*Cryptosporiopsis* cf. *quercina*	*Triptergyium wilfordii* Hook. f.	Pathogenic fungi	Produce cryptocin and cryptocandin toxic to pathogens *Pyricularia oryzae*	Strobel et al., 1999
*Penicillium chrysogenum* Pc_25*, Alternaria alternata* Aa_27	*Asclepias sinaica* (Bioss.)	Pathogenic microorganisms	Synthesizing extracellular enzymes viz., amylase, pectinase, xylanase, cellulase, gelatinase, and tyrosinase.	Fouda et al., 2015
*Talaromyces trachyspermus*	*Withania somnifera* (L.)	Phytopathogenes	Via antagonistic activity to pathogens and enhancing IAA, phosphate solubilization, and siderophore synthesis	Sahu et al., 2019
*Diaporthe* sp. CEL3, *Curvularia* sp. CEL7	*Chloranthus elatior* Sw.	Pathogenic fungi	Synthesized volatile and non-volatile compounds, soluble antifungal metabolites	Santra and Banerjee, 2023
*Pestalotiopsis* sp.*, Neopestalotiopsis parvum and Hypoxylon investiens*	*Taxillus chinensis* (DC.) Danser	Pathogenic fungi	Antifungal activity	Song et al., 2023
*Enterobacter, Microbacterium, Pseudomonas, Rhizobium, and Streptomyces*	*Viola odorata* L.	Phytopathogenes	Synthesis of antimicrobial and antioxidant products, free radical scavenging capacity	Salwan et al., 2023

These studies confirm that endophytes may increase the hosts’ tolerance to pathogens through diverse methods. In summary, while endophytes invade plant tissues, they impact the interactions between both the endophytes and the pathogens, perhaps causing facilitation (positive stimulation of pathogens), negatively reinforcing host resistance, or exhibiting merely no effect (Suryanarayanan et al., 2009; Adame-Alvarez et al., 2014; Schmidt et al., 2014). Nevertheless, it is unclear how endophytic entomopathogenic fungi invade and are colonized; this requires additional research for confirmation. Plants sense the information signal of stresses and respond accordingly to activate specific molecules to combat such stressors. Furthermore, the behavior of a given plant species or cultivar may vary, plant responses are frequently organ-dependent, and findings acquired with whole plants are sometimes misleading.

## 6 Mechanisms mediating plant-microbe interactions to alleviate biotic-abiotic stresses

**Figure 3:**
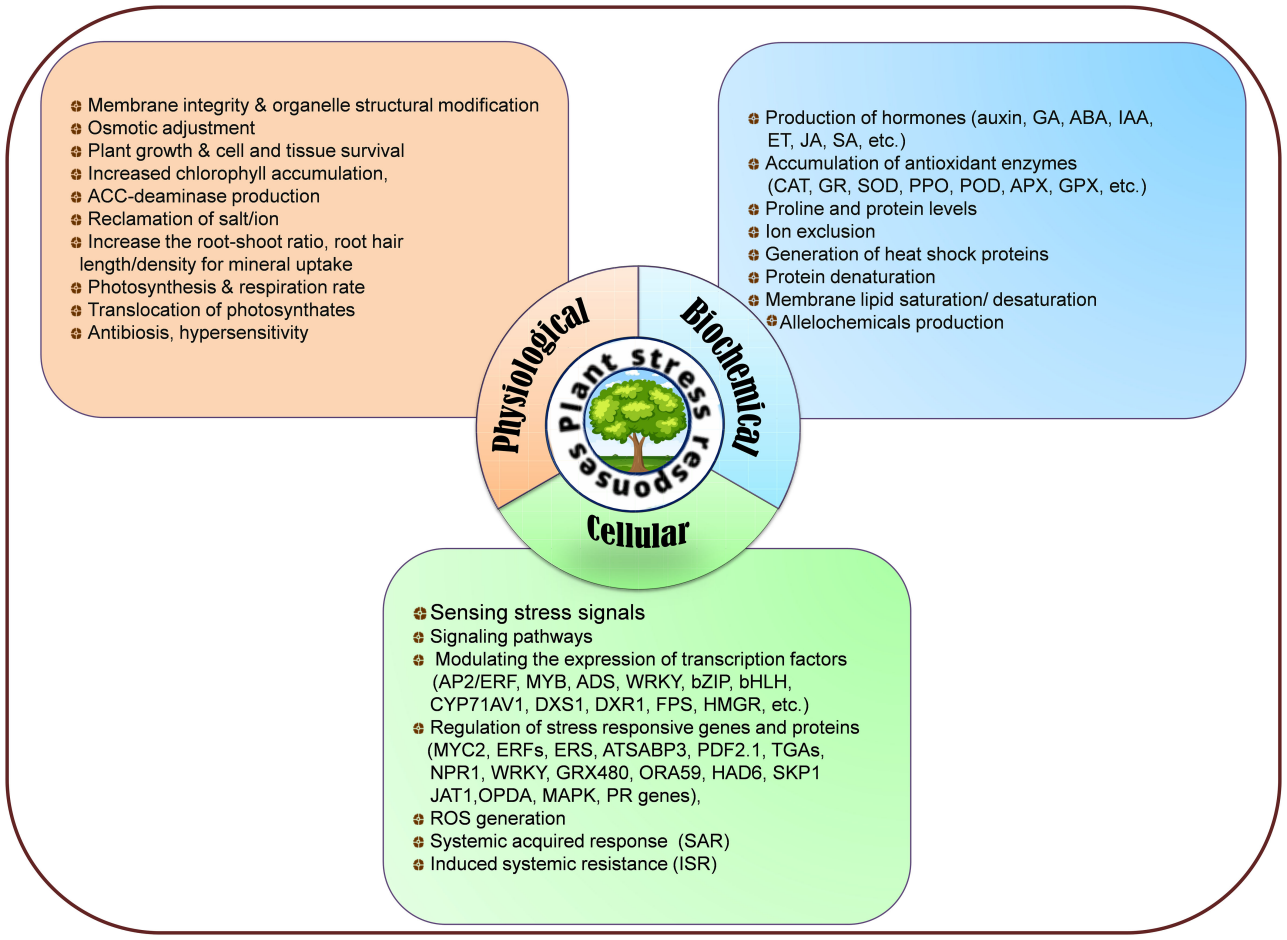
Physiological, biochemical, and cellular responses to mitigate biotic and abiotic stresses.

Plants have developed a multitude of physiological (membrane integrity, organelle structural changes, osmotic adjustments, photosynthesis, and respiration, cell and tissue survival, reclamation, increased root-shoot ratio, increased root hair length and density, photosynthates translocations, antibiosis, hypersensitivity, etc.), biochemical (phytohormones synthesis, proline, protein levels, increased chlorophyll accumulation, ACC-deaminase production, antioxidant enzymes accumulation, ion exclusion, generation of heat-shock proteins, protein denaturation, membrane lipid saturation/unsaturation, synthesis of allelochemicals. etc.), and cellular (sensing of stress signals, signaling pathways, ROS generation, SAR, ISR, modulating expression of stress-responsive genes and proteins, regulation of transcriptional factors, etc.) adaptive mechanisms to withstand stressful environments (**Figure 3**). Endophytes live close interactions with plants and penetrate host plants through their roots, seeds, leaves, and stems to colonize their internal tissues. During the initial phases of colonization, endophytes produce exopolysaccharides (EPS), which aid in adhesion to the root surface and shield them from oxidative damage (Wan et al., 2012). During the fungal transmission of phosphate and nitrogen, the AMF mycelial system mainly spreads around plant roots and facilitates nutrient intake that promotes plant growth in adverse circumstances. Moreover, by maintaining plants’ homeostasis, endophytes diminish water stress damage and trigger regulons like DREB2, stress-induced gene expression, better CO_2_ fixation, starch and phenolics, HSPs generation, balancing carbohydrate metabolism, disrupting plasmalemmas, and reinforced cell walls to face of drought and temperature (heat and cold) and strengthen the functioning of protective enzymes and osmosis delivering plants more resilience plants to various abiotic stressors including drought, waterlogging and salinity (Barka et al., 2006; Nakashima et al., 2012; Raza et al., 2021a). Different strategies for enhancing salt stress tolerance triggered by microbial inoculation are synthesis of antioxidant enzymes, phytohormones, ACC-deaminase, volatile organic compounds, osmoprotectant compounds (glycine, proline, alanine, glutamic acid, threonine, serine, choline, betaine, aspartate, and organic acids), altering ion transporters, resulting in water, ionic, and osmotic homeostasis. They further strengthen plant resistance to heavy metal toxicity through transport, cell wall development, redox communication, and intra/extra-cellular trapping. Most of these abnormalities in reaction to stressful situations are attributed to the creation and dissemination phytohormones in plants’ subterranean and aerial parts (Verma et al., 2016; Arif et al., 2021). Phytohormones also operate as signal molecules between endophytic microbes and plants, regulating structural and morphological changes necessary for plant growth and to accelerating total root biomass through expanding root length and surface (Spaepen et al., 2007). For instance, *Sphingomonas* sp. isolated from *Tephrosia apollinea* augment host plant growth through IAA production (Khan et al., 2014), *Pseudomonas spadiceum* lowers osmotic stress by producing GA (Waqas et al., 2012) and *Pseudomonas*, *Sphingomonas*, *Stenotrophomonas*, and *Arthrobacter* sp. generate cytokinins that perform an indispensable function in plants including apical dominance, chloroplast development, cell growth and transformation, senescence prevention, and plant-pathogen interactions (De Hita et al., 2020). Endophytes, including *Rhizobium* sp., *Azospirillum brasilense*, *Burkholderia cepacia*, *Acetobacter diazotrophicus*, and *Klebsiella oxytoca* have the potential of biological nitrogen fixation that supply alternate nitrogen for farming (Kong and Hong, 2020). Additionally, some endophytes, such as *Pseudomonas fluorescens* have the potential to dissolve insoluble phosphates or to liberate organic phosphates through the manufacturing of citric, malic, and gluconic acids (Otieno et al., 2015). Endophytes are also successful in bioremediation (Ayilara et al., 2023) through various methods, such as reducing heavy metal stress (Zhang et al., 2012) and removing dangerous greenhouse gases (Stępniewska and Kuźniar, 2013). In heavy metal-contaminated soil, bacterial root endophytes associated with the medicinal plant *Festuca rubra* produce siderophores (hydroxamate and catechol) that accelerate host plant development (Grobelak and Hiller, 2017).

Biocontrol strategies by endophytic microbes exist directly through pathogen control or indirectly utilizing systemic plant resistance (Santoyo et al., 2016). They produce different kinds of siderophores (phenolate, hydroxamate, carboxylate, etc.) to converse security against pathogens (Rajkumar et al., 2010). Competition for habitats and food resources, the formation of cell wall-degrading enzymes, lytic enzymes, antibiotic compounds, the commencement of ISR, and the quenching of pathogens’ quorum sensing, among some of the other mechanisms (Rajesh and Rai, 2014). The majority of endophytes are recognized for synthesizing secondary metabolites, notably phenols, terpenoids, alkaloids, flavonoids, steroids, and peptides, which have potent antifungal and antibacterial effects and restrict the spread of harmful pathogens. There have been numerous reports of endophytes producing a variety of lytic enzymes, including chitinase, amylase, proteases, cellulose, and hemicelluloses (Bodhankar et al., 2017). Lytic enzymes are critical for establishing endophytes in host cells by the formation of protein biofilms as well as polysaccharides, which lend phytopathogens’ cell walls structural rigidity (Limoli et al., 2015). Nevertheless, it is also beneficial in managing plant diseases through cell wall breakdown while causing cell death (Cao et al., 2009). The virulence-associated factors, viz., biofilm creation, toxin synthesis, antibiotic resistance, and secretions of degradative exoenzymes, are closely governed by quorum sensing. Several pathogenic microbes, *Pseudomonas* and *Ralstonia*, effectively employ acylated homoserine lactones for communication, causing significant crop damage (Mansfield et al., 2012). In order to prevent infection, the antiquorum sensing mechanism could be employed (Chen et al., 2013). Moreover, once a pathogen attacks, the inherent immune system is triggered, which blocks the pathogen’s invasion and stops its spread. It is an early defense system against phytopathogens, which involves physical barriers like trichomes, stiff cell walls, and waxy cuticles. Plants release exudates from their roots, comprising proteins, amino acids, and organic acids, which interact among the host plant and endophytes (Kawasaki et al., 2016; Shen et al., 2019; Inbaraj, 2021). Hyperparasitism is a novel biocontrol mechanism where the parasitic host is a plant pathogen; probably the most common hyperparasite is a well-known necrotrophic mycoparasite called *Trichoderma* species that feeds on host mycelium (Qualhato et al., 2013).

**Figure 4:**
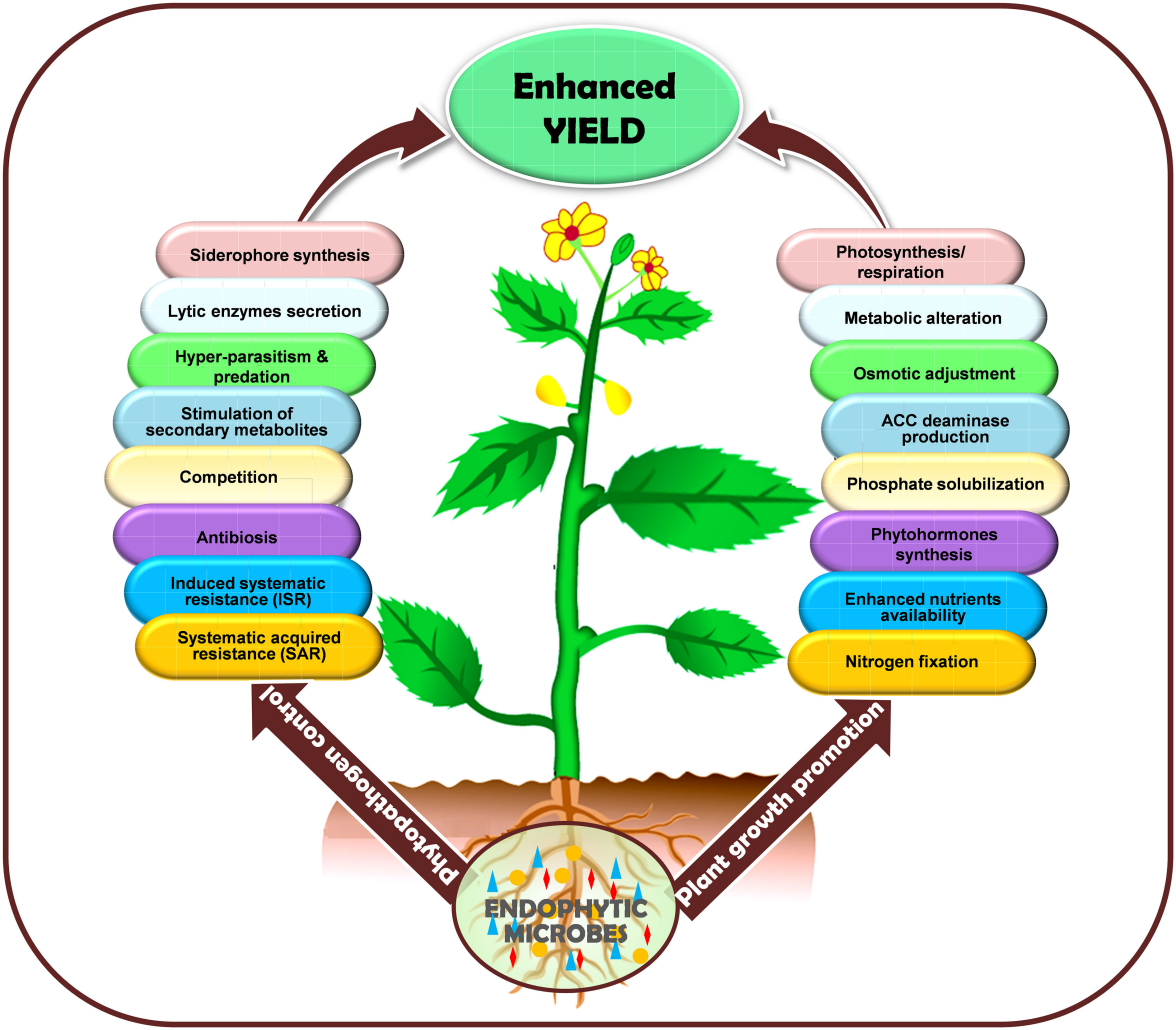
Schematic representation of the endophytes mediated mechanisms in biotic-abiotic stress amelioration in plants. The figure depicts endophytes boosting crop yield through enhancing abiotic stress tolerance by promoting plant growth via regulating nutrient supply and metabolism, phytohormones, osmotic adjustment, photosynthesis, and respiration rate while controlling biotic stress (phytopathogens) through antibiosis, SAR, ISR, competition with pathogens, hyperparasitism, and synthesizing toxins.

In summary, plant-microbe interactions are an efficient, eco-friendly way for plants to cope with severe environmental conditions. Plants evolved multifaceted relationships with diverse groups of microbes to combat biotic-abiotic stresses. Generally, microbes stimulate plant growth by optimizing the physiology and metabolism of the host through different mechanisms. The symbiosis relationships of microbes on host plants might encourage their recruitment through responsive feedback regarding plant health. Endophytes strengthen crop yield by promoting plant growth via regulating nutrient supply and metabolism, enhancing abiotic stresses (heat, drought, waterlogging, salinity, metal-toxicity etc.) tolerance by generating phytohormones, osmotic adjustment, photosynthesis, and respiration rate while controlling biotic stresses (phytopathogens) through antibiosis, SAR, ISR, competition with pathogens, hyperparasitism, and synthesizing toxins and currently extensively utilized in sustainable agriculture. The mechanism strategies whereby endophytic microbes promote plant growth and control phytopathogens, resulting in increased yields, have been schematically illustrated in **Figure 4**.

## 7 Hormonal signaling and crosstalk to mitigate biotic-abiotic stresses

Plants’ defense mechanism is influenced by many factors, primarily genetic makeup and the physiological condition of the plant. Each cell in a plant’s defense system has figured out how and where to respond to stressors, thereby creating an inherent immunity. Among these strategies, phytohormones substantially impact plants’ ability to endure stresses. Generally, cytokinins, gibberellins (GAs), and auxins (IAAs) are linked to plant growth and development, whereas ET, JA, and SA are related to plant defense (Koo et al., 2020; Hossain et al., 2021). GAs and IAAs play a significant role in abiotic and biotic stress tolerance, whereas ET, JA, and SA promote abiotic stress tolerance (Kazan, 2013; Santino et al., 2013; Colebrook et al., 2014). When carried directly to the appropriate cells or transmitted to distant tissues, these hormones influence various physiological networks at low concentrations, increasing resistance to environmental stresses (Colebrook et al., 2014). A comprehensive phytohormone network’s tweaking enables plants to respond in a balanced way to developmental and environmental stimuli.

### 7.1 Ethylene signaling

ET, the gaseous phytohormone, has diversified functions in plants, including cell division and elongation (Love et al., 2009), apical dominance (Yeang and Hillman, 1984; De Martinis, 2000), senescence and abscission (Pierik et al., 2006), flowering (Ogawara et al., 2003; Wang et al., 2013), fruit ripening (Barry and Giovannoni, 2007), breaking seed dormancy and promoting seed germination (Corbineau et al., 2014; Wang et al., 2018; Ahammed et al., 2020), as well as a critical role in programmed cell death (Bouchez et al., 2007). It is a crucial player in both harmful and advantageous plant-microbe interactions (Pierik et al., 2006; Schaller, 2012; Ravanbakhsh et al., 2018; Liu et al., 2019), either through interactions with other phytohormones (Leon-Reyes et al., 2009; Leon-Reyes et al., 2010; Zander et al., 2010) or by controlling the expression of ethylene-responsive genes (Broekaert et al., 2006; Teixeira et al., 2019). Since many biotic and abiotic perturbations influence plants’ physiological and developmental processes, ET synthesis plays a pivotal role in the plant’s adaptation to these environmental threats (Arraes et al., 2015; Sun et al., 2016; Fröhlich et al., 2023). The sensing of ET signaling occurs at the endoplasmic reticulum membrane, triggering a signaling cascade that controls the transcription of ethylene-responsive genes in the nucleus via ERFs (ethylene-responsive factors) (Ju and Chang, 2015). However, the ET-signaling pathway in *Arabidopsis* is negatively regulated by the ET-receptors viz., ethylene response sensors (ERS1, ERS2), ethylene response (ETR1, ETR2), and ethylene insensitive4 (EIN4) (Liu and Wen, 2012). These ET-receptors stimulate constitutive triple response1 (CTR1) in the absence of ET-signaling, which restricts EIN2, a positive regulator of ET-signaling, through phosphorylating EIN2’s C-terminus. Conversely, the presence of ET renders the ET receptors inactive, thereby preventing CTR1 activation. Subsequently, dephosphorylated and cleaved EIN2 C-terminus (CEND) reaches the nucleus, where it stimulates the function of ethylene-insensitive3/ethylene-insensitive3-like1 (EIN3/EIL1), which modulates the expression of ethylene-responsive genes like ERFs. ERFs constitute transcription factors (TFs) with AP2domains that control various genes associated with stress tolerance, growth, development, and hormone-related pathways (Chen et al., 2010; Shakeel et al., 2015; Zhao et al., 2021).

The up-regulation of ET-biosynthesis genes following interactions with advantageous microbes reveals that ET-signaling is activated not only in response to pathogenic microbes but also to helpful endophytic microbes before they are recognized as friends, possibly to optimize the colonization of adequate levels of beneficial microbes (Ravanbakhsh et al., 2018; Eichmann et al., 2021). Owing to inherent physiological reactions to abiotic stressors, plants can instantly produce an enormous amount of ET, which helps the plants to withstand external challenges, but it can also jeopardize growth and development, thereby reducing crop yield and productivity since increased ET levels can cause senescence, abscission, and chlorosis. Research on plant growth-promoting rhizobacteria (PGPR) has shown that they can prevent soil-borne pathogen infections in plants in an ET-dependent way. Furthermore, beneficial microbes can stimulate ISR and SAR in plants to control diseases (Ton et al., 2001).

### 7.2 Salicylic acid signaling

SA, a key phytohormone, has crucial physiological and cellular impacts on plants, including membrane permeability and photosynthetic metabolism, and absorption and transport of ions during stress (Noreen et al., 2009). Furthermore, SA is recognized to outwit various abiotic stresses like ROS, pathogens attacks, drought, and salinity (Hara et al., 2012). Additionally, it regulates plant responses to infection by diversified pathogens, viz., bacteria, fungi, viruses, etc. (Fujita et al., 2006; Loake and Grant, 2007), and is necessary for developing resistance strategies like host cell death, ISR, and SAR. The expression of various genes, including those encoding PR-proteins (pathogenesis-related proteins), might be a mechanism whereby SA induces stress tolerance (Nakashima et al., 2009). The cytoplasm contains an oligomer of *NPR1*, a crucial regulator of SA-induced plant resistance. Once a disease has occurred, it monomerizes and transports to the nucleus, activating a series of genes involved in pathogenesis (Kinkema et al., 2000). But in normal plants, Cys156’s S-nitrosylation, which prevents its monomerization, controls the oligomer to monomer switch. Following infection, nitrous oxide (NO) accretion causes the *Arabidopsis thaliana* SA-binding protein 3 (ATSABP3) to become S-nitrosylated at Cys280, which reduces the protein’s capacity to bind to SA and inhibits its carbonic anhydrase function (Wang F. et al., 2019). In contrast, S-nitrosylation regulates SAR by focusing on the *NPR1/TGA1* system. As mentioned earlier, SA activates thioredoxin (*TRX*), which helps denitrosylate *NPR1* so that it may be monomerized throughout the plant immune response (Kneeshaw et al., 2014). This facilitates *NPR1* to enter the nucleus and interact with the primary leucine zipper transcription factor *TGA*, which in turn makes it easier for *TGA* to bind to the gene-expression promoters. Upon sensing and detecting stimuli of stresses, mitogen-activated protein kinase (MAPK) cascades are triggered that regulate the stress-modulatory systems and are responsible for the signaling of diverse cellular activities under different stressors (Brader et al., 2007). SA facilitates the activation of MAPK pathways driven by pathogen infection and the subsequent production of PR genes for host defense (Xiong and Yang, 2003). Following MPK3 phosphorylation, the *Arabidopsis* protein VIP1 is translocated into the nucleus and functions as a covert inducer of *PR1* genes (Pitzschke et al., 2009). Similarly, MAPKs such as MPK3, MPK4, and MPK6 are confronted with different stresses (Ichimura et al., 2000; Gudesblat et al., 2007). Moreover, pathogen-associated molecular patterns (PAMPs), such as flagellin, activate MAPK cascades to develop pathogen response signaling (Chinchilla et al., 2007). In addition to interacting with ABA-signaling pathways and ROS to improve plant defense, MAPK cascades also play a crucial role in modulating cross-tolerance (Miura and Tada, 2014; Zhou et al., 2014).

### 7.3 Jasmonic acid signaling

JA is another hormone crucial for eliciting responses against various biotic and abiotic perturbations by triggering plant defense signaling systems (Berendsen et al., 2012; Broekgaarden et al., 2015; Wang J. et al., 2020; Yadav et al., 2021). It is ubiquitously present in plants, having multiple regulatory functions, notably root growth inhibition (Han et al., 2023), axis elongation and root formation (Huang P. et al., 2019), leaf senescence (Wang T. et al., 2020), stomatal opening (Suhita et al., 2003), and flower formation (Niwa et al., 2018). Research findings have shown that JAs boost plant growth and development and various adverse environmental circumstances using JA-signaling pathways. Microbe-associated molecular patterns (MAMPs), damage-associated molecular patterns (DAMPs), and herbivore-associated molecular patterns (HAMPs), which are predominantly derived from attacking organisms, cell damage, and abiotic stresses, are some plant-environment interaction models linked to JA-signaling pathways (Newman et al., 2013; Basu et al., 2018; Hou et al., 2019). The most functional JAs in plants’ cells is jasmonyl isoleucine (JA-Ile); however, under normal conditions, its concentration is relatively low (Fonseca et al., 2009). It is recognized that the formation of JA-Ile in plant leaves during stressful situations serves as a physiological defensive system. Jasmonates are transported to the apoplast and nucleus from the cytoplasm by JA-transfer protein1 (JAT1), located in both cell and nuclear membranes (Wang Y. et al., 2019). Even in distant regions, the presence of JAs in the apoplast triggers the JA-signaling system, and the signals are sent to neighboring cells via the vascular bundles and air transmission (Thorpe et al., 2007). Different JAs synthases are localized in the sieve component of vascular bundles, which enables the re-syncretization of JAs throughout their movement (Heil and Ton, 2008). The biosynthesis of the JA precursor 12-oxo-PDA (*OPDA*) in the phloem sieve component has confirmed the theory of re-synthesis. Owing to the reduced level of JA-Ile under normal situations, specific transcription factors (TFs) are unable to activate the promoters of jasmonates-responsive genes. Owing to the reduced level of JA-Ile under typical conditions, specific transcription factors (TFs) cannot trigger the promoters of jasmonates-responsive genes.

The expression of the jasmonates sensitive genes is inhibited by the efficient transcriptional repression complex, composed of the proteins rendering and the putative JAZ (jasmonate-zim domain) interactor. This complex is further activated by histone deacetylase 6 (*HAD 6*), which closes the open complex (Hause et al., 2003). Thirteen JAZ proteins from *Arabidopsis* have been identified to contain the main ZIM domain and the C-terminal JA-associated domain. Different parts of JAZ proteins promote protein complexes (Gimenez-Ibanez et al., 2015). JAZ links with TFs and NINJA (novel interactor of JAZ) [comprising ethylene-responsive element binding factor associated with amphiphilic repression (EAR) motif and recruits TPL (topless)] to form the JAZ-NINJA-TPL repressor complex (Pauwels and Goossens, 2011). The amino acid sequence, JAZ degron, known as JAZ degron seems to have a bipartite structure with a loop and amphipathic alpha hexyl that bind coronatine or JA-Ile and coronatine insensitive 1 (COI1), respectively (Sheard et al., 2010). SKP1 (Suppressor of kinetochore protein1) and SCF (cullin-F-box) create the ubiquitin-proteasome complex. Establishing an SCF-type E3 ubiquitin ligase is the outcome of the interaction between SKP1 and cullin with the F-box protein. In stressful conditions, this F-box protein COI can identify the JA-Ile and deliver it to the nucleus. JA-Ile facilitates JAZ and COI1 communication inside the SCF complex, with inositol pentakisphosphate functioning as a cofactor in the formation of the CO1-JAZ co-receptor complex (Mosblech et al., 2011). JAs-mediated defenses are modulated by the proteasome-mediated degradation of the JAZ protein and the release of transcription factors (TFs) under environmental perturbations. According to Qi et al. (2011), there is solid proof that the expression of the genes that respond to jasmonates is primarily dependent on the linkage of transcription factors (TFs) with JAZ repressors.

### 7.4 Crosstalk between ethylene, jasmonic and salicylic acid

Hormonal signaling crosstalk triggers plants to develop certain specific traits that make them tolerant against the plethora of biotic and abiotic stresses via distinct molecular pathways with a complex network of regulatory interactions (complementary, antagonistic, and or synergistic). Specifically, ET modulates plant defense by controlling the levels of JA and SA (Leon-Reyes et al., 2009; Zander et al., 2010). In such defense responses, ET and JA act synergistically (Penninckx et al., 1998; Zhu, 2014), nevertheless, it has also been reported that they mutually antagonize functions of each other in some specific circumstances (Turner et al., 2002; Bodenhausen and Reymond, 2007). Lorenzo et al. (2003) documented that the ERFs integrate signals from ET and JA. Eventually, other prominent genes that are expressed following the detection of ET and JA include *PDF1.2*, *POTLX3*, *ACS* (ethylene synthesis gene), *THI2.1* (thionin), *PR-3* (chitinase), *PR-4* (hevein-like protein), *PR-6* (proteinase inhibitor), and *PR-9* (peroxidase) (Kolomiets et al., 2000; Norman-Setterbald et al., 2000; Kondo et al., 2007; Chen et al., 2009). However, ET shows antagonistic effects with SA, and they can both suppress each other’s biosynthetic pathways. The direct interaction between *NPR1* and *EIN3* prevents the transcription of genes activated by EIN3, a crucial element of SA signaling (Huang P. et al., 2019). As a result, *EIN3* and *EIL1* bind directly to the SID2 promotor, decreasing pathogen-induced SA production and increasing disease susceptibility in host plants (Chen et al., 2009).

**Figure 5:**
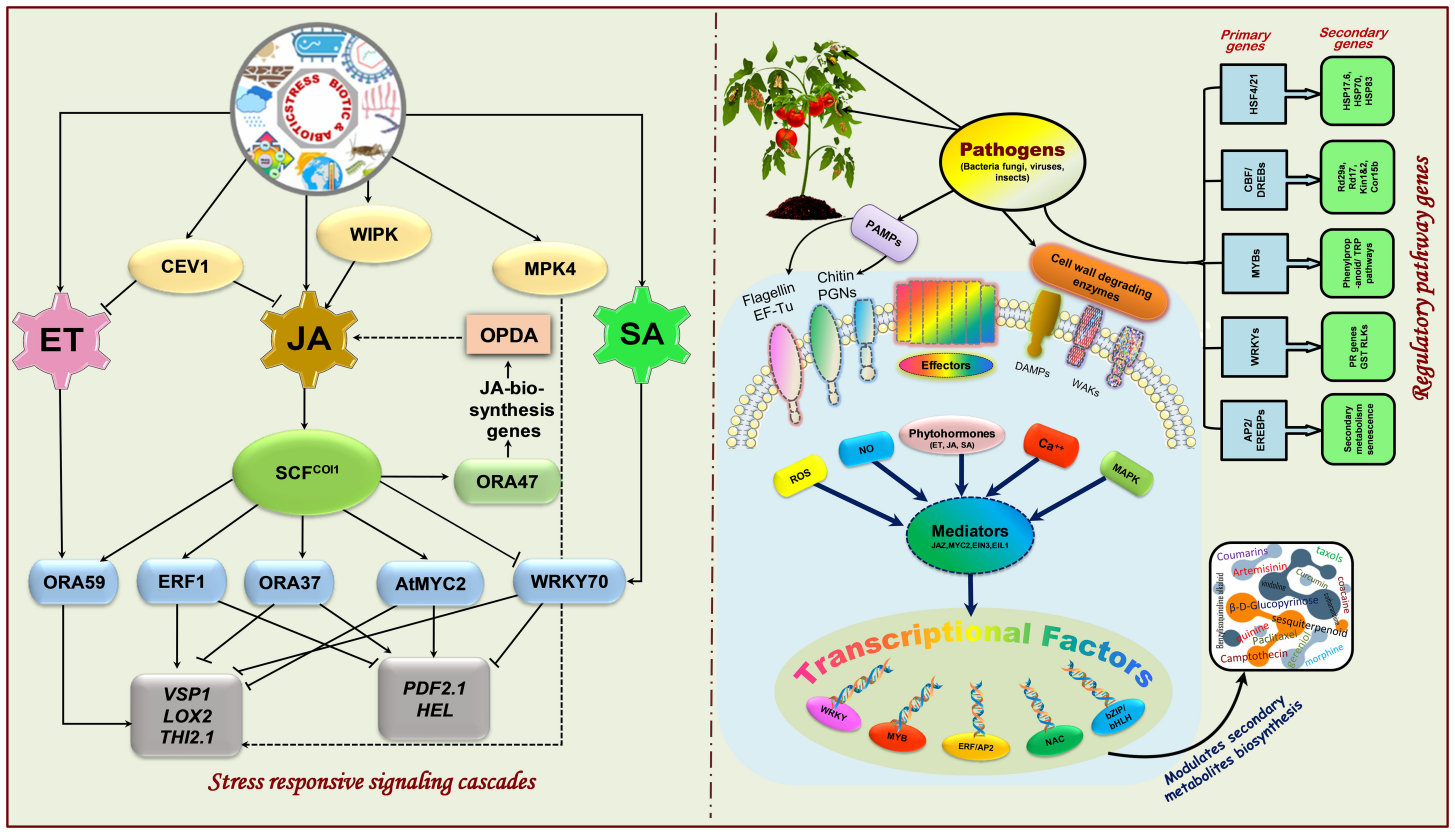
Signaling pathways and regulatory genes to mitigate biotic and abiotic stresses. This figure show a simplified depiction of biotic/abiotic stress-induced signaling pathways like jasmonic acid (JA), salicylic acid (SA), and ethylene (ET) signal transduction and their cross-talk with each other. JA has a central hub position acting with ET and SA. ET, in turn, primarily regulates SCF biosynthesis, transport, and signaling, which is crucial for establishing other genes, like ORA59, ORA37, ERF1, AtMMYC2, and WRKY70, and activation of downstream signaling genes for resistance to different type stresses. Furthermore, a cascade of early (primary) and late (secondary) genes is activated in response to pathogen and insect-induced damage. Genes of herbivory resistance, plant disease resistance, JAs, and endogenous signaling molecules are not only involved in the pathogen resistance mechanism of plants but also have an apparent defensive effect on necrotrophic pathogens. Significant changes in defensive enzymes and secondary metabolites occur, which play essential roles in plant resistance against pathogens. CEV1, Cellulose synthase family protein; WIPK, wound-induced protein kinase; OPDA, 12-oxo-PDA; SCF^COI1^, (Skp, Cullin, F-box containing complex); ORAs, octadecanoid-responsive Arabidopsis; ERFs, ethylene-responsive genes, AtMYC2, Arabidopsis thaliana MYC2; VSP1, vegetative storage protein1; LOX2, lysyl oxidase-like 2; THI2.1, thionin 2.1; PDF2.1, plant defensin2.1; HEL=, AP2: adipocyte protein 2, EREBPs, ethylene-responsive element binding proteins; MYBs, Myeloblastosis; CBF, C-repeat binding factors; DREBs, dehydration responsive element binding protein; HSF4/21, heat shock factor protein, GST, plant glutathione S-transferases; RLKs, receptor-like kinases; TRP, transient receptor potential; Rd, responsive to desiccation, Kln, kallikreins; Cor15b, cold-responsive15b.

Likewise, it is quite interesting that the crosstalk between the antagonistic pathways of hormones JA and SA also results in plant tolerance to various stresses. Several genes, including MYC2, plant defensin 2.1 (*PDF2.1*), TGAs, MAPK, *NPR1*, *ERF1*, *WRKY62*, *WRKY70*, *glutaredoxin 480* (*GRX480*), and octadecanoid-responsive *Arabidopsis* (*ORA59*), play a critical role in JA-SA inter-modulation (Wang et al., 2021). Three NAC (TF family) genes-*ANAC019*, *ANAC055*, and *ANAC072* interact with MYC2 in different ways to prevent SA accumulation. These TFs also regulate the expression of genes that produce SA. *GRX480* preferentially binds to TGAs, modulating *PR1* gene expression, and MPK4 controls *GRX480* positively (SA-signaling pathway), while *MYC2* is negatively regulated (JA-signaling pathway). However, *GRX* genes can prevent the activation of the JA response gene *ORA59* (Wang et al., 2020). The hormonal changes between interactions of JA and SA enhance plants’ tolerance against chilling, drought, and oxidative stress. Methyl jasmonate (MeJA) possesses excellent permeability to cell membranes than JA and is very volatile by nature, and it might quickly diffuse nearby plants (Munemasa et al., 2011). External MeJA supplementation controls the formation of ROS and the immune systems by promoting antioxidant enzyme activity in *Panax ginseng* (Wahab et al., 2022). Following stress sensing, plants rapidly generate ROS (Wojtaszek, 1997; Foyer and Noctor, 2005). Furthermore, the plant meticulously regulates ROS synthesis to prevent tissue damage (Vinocur and Altman, 2005; Mittler et al., 2011; Bhattacharjee, 2008). It has been recognized that although higher levels of ROS are toxic and harmful to organisms and can cause permanent cell death, its lower levels are primarily responsible for controlling stresses. Perhaps ROS could be the critical factor facilitating cross-tolerance between biotic and abiotic stress-responsive stimuli (Choudhury et al., 2013; Kissoudis et al., 2014). A diagrammatic representation of ET, JA, and SA signaling cascade and pathway genes for biotic and abiotic stress tolerance is illustrated in **Figure 5**.

## 8 Endophytic microbes as biostimulants in sustainable agriculture

### 8.1 Benefits

Endophytes are an array of ubiquitous microorganisms that inhabit different niches in plant tissues. In addition to the fact that endophytic microbes can help plants to lessen the negative effects of abiotic stresses, research has shown that endophytes have functional traits with linked detrimental impacts of environmental factors on the continued existence and development of susceptible plant species by synthesizing bioactive compounds, triggering resistance that results from gene expression, and altering the metabolism of certain enzymes. They can inhibit the growth of phytopathogens via the production of antifungal compounds, thereby augmenting crop yields by facilitating plants to acquire nutrients while synthesizing phytohormones. Moreover, they reduce heavy metal stress, eliminate hazardous greenhouse gases, and degrade PAHs in the bioremediation process (Stępniewska and Kuźniar, 2013). Additionally, in recent years, endophytes have gained more recognition for their use in the phytoremediation of a range of environmental pollutants and could be helpful in developing effective cleanup systems (McGuinness and Dowling, 2009; Weyens et al., 2009; Segura and Ramos, 2013; Anyasi and Atagana, 2018; Adeleke et al., 2022). The diversity of endophytes, their ability for stress adaptation, and their synthesis of metabolites make them an endless supply of novel metabolites that can reduce harmful chemicals in agriculture. To illustrate, several studies have reported the beneficial effects of microbial endophytes on a wide range of medicinal plants, including *Withania somnifera, Artemisia annua, Papaver somniferum, Cymbidium aloifolium*, *Salvia miltiorrhiza*, *Catharanthus roseus, Bacopa monnieri*, *Nicotiana tobaccum, Andrographis paniculata, Chlorophytum borivilianum, Panax ginseng, Panax notoginseng, Curcuma longa, Curcuma wenyujin*, etc. (Meng and He, 2011; Karthikeyan et al., 2012; Wang et al., 2012; Ma et al., 2013; Barnawal et al., 2016; Kumar et al., 2016; Hong et al., 2018; Jayakumar et al., 2019; Sahu et al., 2019; Shah et al., 2019; Jiao et al., 2020; Ray et al., 2021; Zheng et al., 2021; Mei et al., 2023; Salwan et al., 2023; Sharma et al., 2023; Song et al., 2023; Wang et al., 2023; Zou et al., 2023). Thus, unquestionably, these endophytes have demonstrated tremendous potential as a green and eco-friendly alternative for boosting food production in sustainable agricultural systems.

### 8.2 Potential applications

Biostimulants are a class of substances or microbes derived from natural resources that are applied to soil or plants to boost crop yield and quality by stimulating plants’ biological processes or enriching the soil microbiome for better nutrition and stress tolerance. Biostimulants have emerged as a boon for sustainable agriculture because they significantly accelerate the process of agronomic trait advancement in plants without jeopardizing yield, quality, or biodiversity. In recent years, endophytic microorganisms have been thoroughly explored for the possibility of being utilized as biostimulants for minimizing the usage of harmful chemicals in agriculture, thereby fulfilling the WHO’s envisioned sustainable development goals while ensuring food and nutritional security (Omotayo and Babalola, 2020). To exemplify, investigations using endophytic microorganisms have demonstrated their potential roles as biostimulants (Kumar et al., 2015; Wani et al., 2016; Hashem et al., 2017; Vyas et al., 2018; Saia et al., 2021; Tharek et al., 2022), biofertilizers (Arora and Mishra, 2016; Santoyo et al., 2016), biopesticides (Gange et al., 2012; Waqas et al., 2015a; Lugtenberg et al., 2016), and biocontrol agents (Hashem et al., 2017; Halecker et al., 2020; Jiao et al., 2020). Likewise, da Silva et al. (2017) developed an inexpensive and efficient biostimulant formulation made with endophytic diazotrophic bacteria and humic acids that boosts crop production while ensuring the finest use of fertilizers. Considering the practical implications, microbial formulations promote plant growth and development by restoring soil minerals, improving plant nutrient uptake, or making nutrients easily accessible (Bashan et al., 2014; Mishra et al., 2015). In addition, they also affect the host’s other beneficial effects, such as osmotic adjustment, stomatal regulation, shaping root architecture, and adjustment of nitrogen accumulation and metabolism (Compant et al., 2005). Bioinoculants facilitate seed treatment by distributing inoculants evenly over seeds, causing systemic acquired resistance (Ma, 2019), and assisting in bioremediation using a metabolic engineering approach (Dangi et al., 2019). In terms of agrochemical and metal pollutants solubilization, bioabsorption, and mineralization, endophytes have also proven effective in environmental remediation (Gavrilaş et al., 2022). Studies have advanced further the potential implementation of microorganisms as traditional biological control agents (BCAs) by inundating inoculation in plants. Tahir et al. (2017) found that *Bacillus subtilis* volatiles negatively impact *Ralstonia solanacearum’s* physiology and ultrastructure and elicit systemic resistance in tobacco against bacterial wilt. The best characterized and most frequently microbial endophytes in biological control programs are *Beauveria bassiana* and *Metarhizium anisopliae* have antagonistic activities on plant pathogens via an array of mechanisms, including the synthesis of metabolites (volatile compounds, antibiotics, and enzymes), competition, parasitic relationships, triggering systemic resistance by the plant, and improvements in plant growth (Vidal and Jaber, 2015; Vega, 2018; Moraga, 2020; Baron and Rigobelo, 2021). In another study, endophytes frequently assist plants in reinforcing their defense mechanisms by facilitating the stimulation of induced systemic resistance, which occasionally overlaps with those of acquired systemic resistance, considering both of them may foster the growth and development of plants (Busby et al., 2016) and protect against phytopathogens (Chadha et al., 2015). Therefore, implementing microbial formulations as biocontrol or biofertilizers might be an effective alternative to the overuse of agrochemicals. Perhaps the most environmentally and farmer-friendly step toward sustainability might be developing consortia from aspiring endophytic strains from native agricultural fields, resulting in multifaceted bio-solutions.

### 8.3 Challenges

Despite the widespread interest in endophyte research, there are still certain challenges in designing efficient microbial formulations, such as:

- Endophytes are tissue-specific; identifying suitable host plants, their healthy tissues or organs is critical.
- Isolating novel endophytes and investigating the relevant complementary or antagonistic signaling pathways during symbiosis.
- Pecularity of microbial consortia in terms of their modes of action. Some endophytes have aseptic or uncultivable properties, making synthetic cultivation challenging. Therefore, developing new bioengineering systems or modifying traditional isolation methods is crucial.
- The biological constraint still exists even though some endophytes’ facultative nature offers the possibility of continued colonization, provided they can survive in the rhizosphere.
- The interactions of microbial biostimulants with the micro-climate (temperature, pH, water, humidity, nutrients, etc.), host plants (defense system and exudates), and native microbes should also be considered.
- The inoculants’ concentration, functionality, and survivability during storage as well as maintaining sterility, are critical for designing efficient formulations.
- Limitation of biological adjuvants as bio-careers.
- Artificially inoculated endophytes may begin acting as latent pathogens by disseminating toxins through the food chain.
- The potential of exogenously applied endophytic microbes to establish a habitat beneficial to both entities is contingent upon their ability to compete successfully with native microbes. Thus, inoculating crops with consortia rather than a single strain will increase their persistence.
- Licensing/registration of formulations before arriving on the market is complicated.

Screening of endophytic microbes in a greenhouse, either solely or in combined applications, has proven to be efficient in maximizing crop yields. Designing formulations with high microbial concentrations and survivability is crucial for developing potent biostimulants. However, finding the most critical factors and ensuring sterility during the formulation process is challenging because testing every possible combination is not feasible. Therefore, the commercial success of endophyte-based biostimulants requires a comprehensive knowledge of molecular plant-microbe interaction, methods of transmission, and strategies for establishing a symbiotic relationship between the endophyte and host plant. The research efforts aimed at discovering microbial biostimulants are beginning, which might result in significant advancement in this emerging field. In modern agriculture, methods to increase the use of endophytic microorganisms are desired to use these microbes alone or in combination with bioprospecting as bioinoculants in crop systems. The most effective methods for using endophytic microorganisms in agriculture have not yet been identified. However, applying endophytes as seed dressings or directly into the soil is the most frequent and common method utilized by farmers. Meanwhile, the implementation of these endophytes-based inoculations is unsuccessful on field sites owing to issues with the endophytes’ establishment.

Therefore, the manifold characteristics of endophytes make them possible alternatives to harmful agrochemicals, and thus, they are now being utilized more frequently throughout the world. Endophyte-based biostimulants are cost-effective, preserve natural soil microbiota, have few or no hazardous byproducts, enrich soil organic matter, and ensure ecosystem sustainability. Utilizing improved microbial inoculants can be one of the best input components for green farming. Although endophytic microorganisms can be engineered, little is known about their use as bioinoculants in contemporary farming situations. Therefore, more research is required to determine the effectiveness of microbial bio-input for commercialization before these endophytes can be used as bioinoculants to improve soil health and crop yield.

## 9 Conclusion

The yield and quality of medicinal plants are considerably influenced by various edaphic and climatic factors such as soil characteristics, soil microbiota, light, humidity, temperature, drought, salinity, etc. To adapt to a stressful environment, plants acclimatize themselves by modulating the genes responsive to stress, transcriptional factors, and biosynthesis signaling pathways. Furthermore, in stressful conditions, plant defense systems trigger appropriate cellular responses by stimuli from the sensors situated on the cytoplasm or cell surface and transmitting signals to the transcriptional machinery in the nucleus with the help of various signaling pathways. Sustainable production is still a significant challenge; perhaps specific strategies might be helpful in such scenarios as rescue measures like integrating plant-associated microbes into farming systems, supporting agricultural production through various interventions, and mitigating biotic and abiotic perturbations. Utilizing endophytic microbes as biostimulants not only eliminates the need for synthetic inorganic pesticides and fertilizers but also lowers input costs and, more importantly, minimizes the impact of these agrochemicals on vital existing ecological communities. Nevertheless, its practical application suffers some limitations, viz., endophytes are tissue-specific, and tissue type, the host, and the environment mainly influence their functionality. However, the information gap of their multifaceted nature in plant tissues has hampered the advancement of endophyte research in various fields. Furthermore, the underlying mechanisms governing these interactions are still not fully explored; several studies have raised the hope of their potential exploitation of plant-microbe interactions in managing various stresses. Therefore, to promote the practicality of endophyte-assisted biological applications as biostimulants, particularly in the field, comprehensive research is necessitated to demonstrate an insight into the microorganisms in its host medicinal plants. Modern high-throughput genomic studies have revolutionized the field of microbiome research by unveiling the enigmatic realms of endophytism, facilitating the pursuit of endophytes, enabling the sequencing of a broader range of microbes, and enticing a comprehensive examination of microbial ecosystems by taxonomic classification, phylogeny, and evolutionary studies, In the future, advanced omics approaches such as genomics, transcriptomics, proteomics, and metabolomics can support an in-depth knowledge of plant-microbe interactions and stress signaling pathways, leading to its potential exploitation in agriculture for improving yield, quality, and resistance of medicinal plants, drug development, and management of the environment.

## Author contributions

PP: Conceptualization, Writing—original draft preparation, Writing - review and editing, Visualization. AT: Conceptualization, Writing—original draft preparation. SD: Writing - review and editing. KL: Writing - review and editing. TJ: Writing - review and editing. All authors contributed to the article and approved the submitted version.

## Glossary

**Table:**

CAT	Catalase
PPO	Polyphenol oxidase
POD	Peroxidase
ACC deaminase	1-Amino Cyclopropane-1-Carboxylate deaminase
SOD	Superoxide dismutase
AMF	Arbuscular mycorrhizal fungus
MDA	malondialdehyde
POD	Peroxidase activity
GR	Glutathione reductase
ALD	Aldehydes
HSPs	Heatshock proteins
PAL	Phenylalanine ammonia-lyase
STS	Stilbene synthase
SAR	Systemic-acquired resistance
ISR	Induced systemic resistance
ROS	Reactive oxygen species
GPX	Guaiacol peroxidise
NPR1	Non-expressor of pathogenesis-related genes
PR1	Pathogenesis-related protein1
CMV	Cucumber mosaic virus
AsSyn	Asparagine synthetase
Gluc	b-1,3-glucanase
BR-SK1	Brassinosteroid signaling kinase 1
TCAS	Tetra-hydrocannabinolic acid synthase
ZF-HD	Zinc finger-homeodomain
RdRP2	RNA dependent RNA polymerase
GAs	Gibberellins
IAAs	Auxins
ERS	Ethylene response sensors
ETR	Ethylene response
EIN4	Ethylene insensitive4
CTR1	Constitutive triple response1
CEND	Cleaved EIN2 C-terminus
EIN3/EIL1	Ethylene-insensitive3/ethylene-insensitive3-like1
TFs	Transcription factors
PGPR	Plant growthpromoting rhizobacteria
PR-proteins	Pathogenesis-related proteins
THI2.1	Thionin2.1
ATSABP3	Arabidopsis thaliana SA-binding protein 3
TRX	Thioredoxin
PAMPs	Pathogen-associated molecular patterns
MAMPs	Microbe-associated molecular patterns
MAPK	mitogen-activated protein kinase
DAMPs	Damage-associated molecular patterns
HAMPs	Herbivoreassociated molecular patterns
JA-Ile	Jasmonyl isoleucine
JAT1	JA-transfer protein1
OPDA	12-oxo-PDA
JAZ	Jasmonate-zim domain
HAD 6	Histone deacetylase 6
NINJA	Novel interactor of JAZ
TPL	Topless
COI1	Coronatine insensitive 1
SKP1	Suppressor of kinetochore protein 1
SCF	Cullin-F-box
PDF2.1	Plant defensin 2.1
GRX480	Glutaredoxin 480
ORA59	Octadecanoidresponsive Arabidopsis
MeJA	Methyl jasmonate
